# Psychometric properties of the health literacy instrument in Brazil (HLS-EU-BR47)

**DOI:** 10.1186/s12889-024-19108-2

**Published:** 2024-06-20

**Authors:** Luis Saboga-Nunes, Uwe H. Bittlingmayer, Stefanie Harsch, Silvana Ligia Vincenzi, Silvio Aparecido da Silva, Andréa Cristina Konrath, Lizandra Brasil Estabel, Eliane Lourdes da Silva Moro, Luciane Alves Santini, Filipe Xerxeneski da Silveira, Darclé Cardoso, Roselita Sebold, Celeste Aparecida Pereira Barbosa, Marta Regina Farinelli, Dalton Francisco de Andrade

**Affiliations:** 1 Institute of Sociology, University of Education Freiburg, Freiburg, Germany; 2 UNESCO Chair and WHO Collaborating Center in Global Health & Education, Paris, France; 3 Federal University of Santa Catarina, Florianópolis, Brazil; 4 Federal Institute of Santa Catarina, Florianópolis, Brazil; 5 Federal University of Rio Grande Do Sul, Rio Grande Do Sul, Brazil; 6 Programa de pós ?graduação em odontologia em saúde coletiva, Postgraduate Program in Dentistry, collective health, Federal University of Santa Catarina, Florianópolis, Brazil; 7 Federal University of Santa Catarina, Rio do Sul, Santa Catarina, Brazil; 8 Federal University of Triângulo Mineiro, Uberaba, Minas Gerais Brazil

**Keywords:** Health literacy, Item response theory, Gradual response model, Validity of the instrument, measurement scale of health literacy, Brazil

## Abstract

**Background:**

Health literacy (HL) is a key component of health promotion and sustainability and contributes to well-being. Despite its global relevance, HL is an under-researched topic in South America but is now debuting its exploration in Brazil. To leverage its benefits for South America, the mere translation of validated tools into Portuguese is insufficient. Rather, it is necessary to examine their validity. This study aims to assess the psychometric properties of the European Health Literacy Questionnaire (HLS-EU-BR47) using the Item Response Theory (IRT) in a population-based sample of adults in Brazil.

**Methods:**

A cross-sectional online study was conducted across Brazil and included 1028 participants aged 18 years and above (80% women). Cronbach’s alpha, McDonald’s omega, factor analysis, graded responses model, Item Characteristic Curve, HL levels based on this, HL standard calculation, IRT, and regular score correlation were computed.

**Results:**

The instrument exhibit high reliability (Cronbach’s alpha 0.95). Factor analysis yielded one factor. IRT was appropriate for data analysis because it allowed quality evaluation of items and constructed a scale to quantify HL. The 47 items and latent features of respondents in the same unit of measurement are positioned in the construction of the HLS-EU-BR47 instrument. The percentages of individuals at each HL level, calculated using IRT, were found to be comparable to those obtained through the standard computation, e.g., 3.2% of people reported very low HL versus 10.8% inadequate HL, 56.2% reported low HL versus 39.5% problematic HL, 31.1% had moderate HL versus 30.1% sufficient HL, and 9.5% had high HL versus 19.7% with excellent HL. The mean HL scores were comparable between women and men (33.9 vs. 33.7, *P* = 0.36).

**Conclusion:**

This study provides new evidence of the validity of a widely used HL instrument for the population of South America (in this case, Brazil). This tool can be utilized by citizens, health professionals, and regional/national policymakers to inform the development of initiatives to assess and improve the HL of individuals, groups, and communities. Further studies are needed to confirm and extend the findings and to explore the influence of local cultures and practices in the vast Brazilian territory on HL.

**Supplementary Information:**

The online version contains supplementary material available at 10.1186/s12889-024-19108-2.

## Introduction

Health literacy (HL) is a public health goal [1], and its utmost relevance for health promotion, prevention, and healthcare on all societal and ecological levels is now widely recognized [2]. Moreover, the World Health Organization (WHO) recommended tailoring citizens’ HL development to different social conditions linked to sustainability [3] and “well-being societies” [4]. The Geneva Charter for Well-being builds on the Ottawa Charter for Health Promotion [5] and the heritage of ten global health promotion conferences. Two of these deserve mention. The seventh Global Conference on Health Promotion of the WHO, held in Nairobi, Kenya, in 2009, introduced HL as one of five key health promotion strategies [6]. Also, in 2016, the ninth Global Conference on Health Promotion, held in Shanghai in 2016, highlighted the role of HL and linked it closely to sustainable development [3]. In 2021, the WHO reiterated the necessity of HL and assigned it a high priority over the life course [4].

However, in order to effectively promote HL, a comprehensive understanding of HL and the HL levels within the country or target group in question is needed [2]. Additionally, knowing the level of HL at the citizen level will facilitate this development, as it provides the Ministry of Health and health workers with a baseline [7, 8]. However, to assess HL appropriately, an adequate tool must be available. Operationalizing HL has been one of the methodological issues in this field. Several tools to assess HL are available [9], for example, the Test of Functional Health Literacy in Adults (TOFHLA) [10], the Rapid Estimate of Adult Literacy in Medicine (REALM) [11], or the Newest Vital Sign (NVS) [12]. Two characteristics of these tests are their narrow scope, measuring only specific language modes (reading and/or numeracy), and their primary use in healthcare settings, with no focus on health promotion. Although TOFHLA and REALM are the most commonly used HL tools [13], other tools have recently been introduced in HL research, such as the National Assessment of Adult Literacy Survey [14], the Swiss Health Literacy Survey [15], the Health Literacy Instrument for Adults (HELIA) [16], the Health Literacy Questionnaire (HLQ) [17], and the HLS-EU-Q developed for the European Health Literacy Survey [7]. A plethora of instruments have been developed in recent times that cannot be exhaustively catalogued here. For a comprehensive list of HL tools, please refer to the HL toolshed [18]. Of these, the HLS-EU-Q is the most comprehensive, as it explores four dimensions of HL: the ability to find, understand, appraise, and apply health information in three domains: health care, disease prevention, and health promotion [19].

The HLS-EU-Q has been established over time through research involving multiple cultures, allowing for the first simultaneous assessment of citizens’ HL in the world in different social contexts, including various European and Asian countries [7, 8, 20]. Despite its extensive use, studies that assess HL in South America with this instrument are lacking. Consequently, there is a paucity of data regarding the HL level in South America, particularly with regard to HL levels in the domains of health promotion and disease prevention. A review of studies conducted worldwide has revealed that the knowledge of the low levels of HL among their populations has prompted the implementation of more health interventions. Given that Brazil is the seventh largest country in the world with a population of 217.24 million and pressing health needs, it is of the utmost importance to assess its HL and develop targeted interventions. Such an assessment would assist Brazil in fulfilling the promise made at the 9th and 10th Global Conference on Health Promotion to improve public health through HL.

The first step is to measure HL of the Brazilian population, rather than limiting the assessment to selected subgroups, such as older people, patients with diabetes [21, 22]. However, to do so, not every questionnaire is equally suitable for this purpose in the given context. Therefore, it is necessary to assess the validity of the questionnaire prior to its use. Various well-established instruments, including the HLQ, the TOFHLA, or the short TOFHLA [23–25] can be used, translated, and even culturally adapted here. The objective of our study was to assess comprehensive HL, encompassing all domains of the health spectrum (from health promotion to disease management) and all dimensions from finding, understanding, assessing, to applying. Consequently, we decided to use the HLS-EU-Q47. The HLS-EU-Q47 has been employed in a multitude of studies across the globe, thereby demonstrating its global relevance and facilitating a comprehensive understanding of HL [8, 26, 27]. However, the measurement of HL is only as reliable and accurate as the instrument itself. For this purpose, it is necessary not only to check the instrument’s reliability but also to conduct a full validation, for example, using Item Response Theory (ITR). IRT, also known as latent response theory, has been successfully applied [28–30] in different scientific fields including education, psychology, administration, health sciences, psychology, and engineering) [29, 31–35, 36]. IRT encompasses models for the assessment of latent traits. A latent trait is a characteristic of a respondent that cannot be directly observed, that is, no instruments can measure it directly. In this investigation, the latent trait of interest is the HL.

IRT models explore the pathways to represent the relationship between a respondent’s latent trait in the specific knowledge domain being evaluated or verified with the likelihood of she or he providing a particular answer to an item. IRT emerged in the mid-1950s and was developed to overcome some limitations of Classical Test Theory (CTT) [37, 38]. It is regarded as an advancement of classical psychometry, complementing and enhancing statistical techniques for analyzing for items and scales [38–41]. The advantages of IRT over the Classical Theory of Measures are as follows: IRT positions items and study participants on the same scale, thereby enabling the level of a participant’s trait’s characteristic to be compared to the level of the characteristic required by the item. This enables the interpretation of the constructed scale and facilitates the knowledge about which items provide information along the scale [41]. Another advantage of IRT is that it adheres to the principle of invariance, i.e., the items’ parameters of the items do not depend on the latent trait of the respondent and respondent parameters do not depend on the items presented [39]. A variety of IRT models have been developed, and the selection of a particular model is contingent upon the nature of the item (e.g., dichotomous, polytomous, gradual), the characteristics of the latent trait (accumulative, non-accumulative), and the dimensionality of the latent trait (one-dimensional or multidimensional). Dimensionality refers to the number of latent traits to be analyzed. Most IRT applications concern one-dimensional models [29, 32, 33], although there are also instances of multidimensional models, albeit in smaller numbers. In this research, the Graded Response Model (GRM) of Samejima [42–44] is employed.

The study aims to explore the psychometric properties of the HLS-EU-BR-Q47 measurement tool with ICT and GRM and to assess the HL level in Brazil with the HLS-EU-BR.

## Method

### Research design

In 2011, the research group ProLiSa (PROmoção em comunicação, educação e lIteracia para a sAúde) of the Portuguese Speaking Network for Health Literacy Promotion (Rede Lusófona para a PROmoção da lIteracia para a sAúde) was established with the objective of facilitating the translation of HL knowledge into Portuguese-speaking countries. In 2014, ProLiSaBr was awarded a CNPq research grant in Brazil (dgp.cnpq.br/dgp/espelhogrupo/7,607,450,991,114,518) to raise social and political awareness of the importance of HL in Brazil. This research project follows other ProLiSa initiatives aimed at gaining a deeper understanding of the relevance of HL in this cultural context. The objective was to ascertain the validity of an instrument designed to assess the level of HL at both the individual and community levels. With two distinct poles under the same research flagship, one in Minas Gerais and one in Porto Alegre, the ethics committees approved the respective protocols. Consequently, at the Federal University of Triângulo Mineiro, the CAAE process 04697018.3.0000.5154 originated the approval 3.290.664. At the Faculty of Librarian & Communication of the Federal University of Rio Grande do Sul, under the process code CAAE 45816921.0.0000.5347, received approval number 4.885.152.

#### Sample selection and data collection

In order to ensure high variability and good representativeness of the sample, we opted for two samples and a more conservative approach for its size characterization. This research follows the principle of purposive sampling, where the data saturation is a key aspect of sample size definition. Once data saturation has been reached, and new information gathering has no impact on the results or conclusions, the number of participants to be included is determined. Accordingly, the recommended minimum sample size for item development of EFA (Exploratory Factor Analysis) is five participants, while the minimum sample size for CFA (Confirmatory Factor Analysis) is ten participants [45]. Given that the instrument in question comprises 47 items, the minimum sample size for CFA would be 500 participants. In a more conservative approach, Hambleton [46] suggests that IRT requires a large sample size (e.g., 1,000) to ensure the accuracy of item-parameter estimates.

**Fig. 1:**
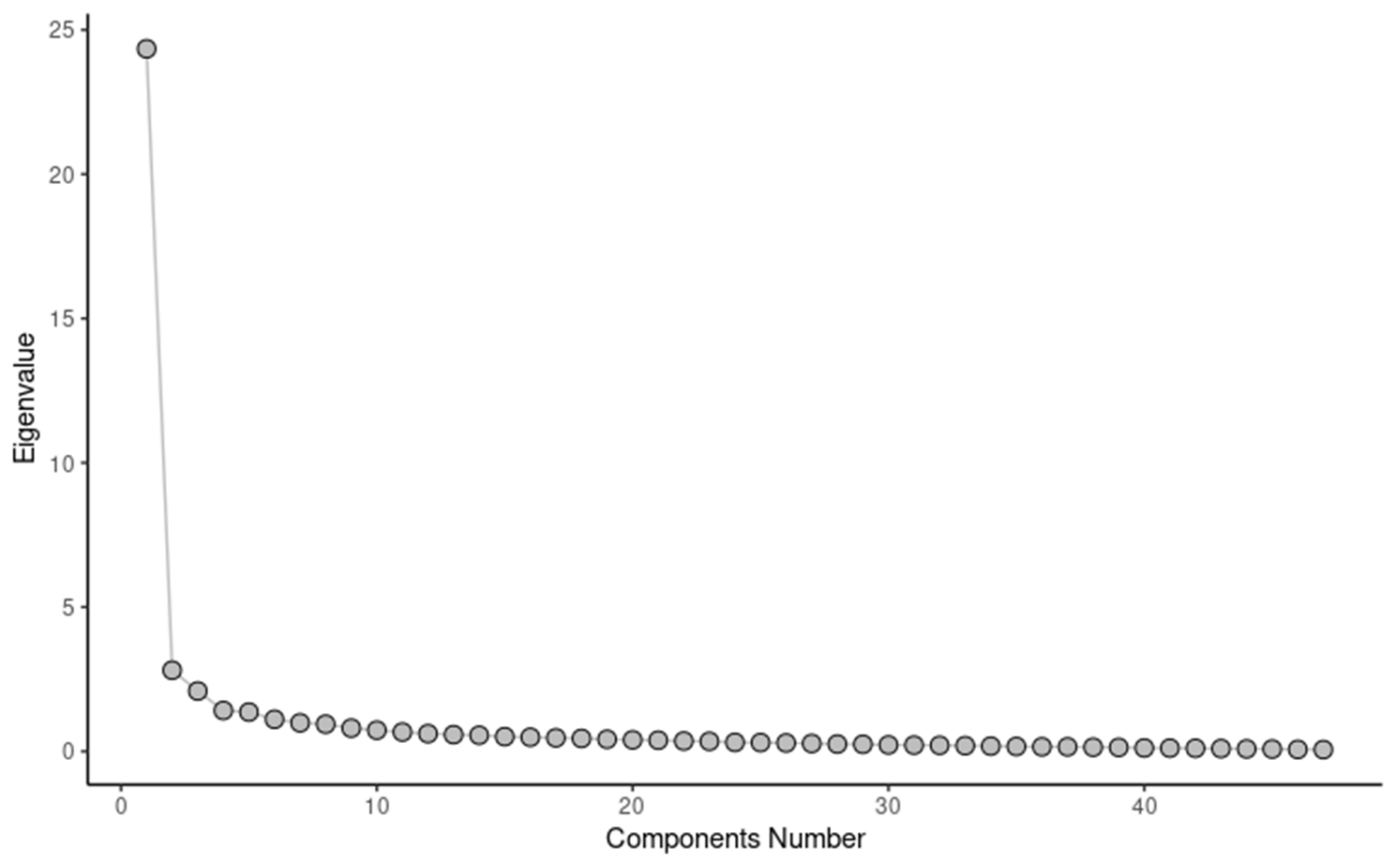
Analysis of the eigenvalues of the polychoric correlation matrix

Therefore, our first sample focused on professional groups, including, health professionals, students of specialization health courses, users of public health services, teachers of the municipal education network, librarians, managers, and users of public services in the state of Minas Gerais. The second sample included representatives across all major regions of the country. In total, 1,028 respondents completed the questionnaire. Upon applying the HLS-EU-Q criteria for the inclusion or exclusion of respondents e.g., if the person does not demonstrate variability in response, they should be excluded), 37 respondents were excluded. the final sample for analysis consisted of 981 respondents, 245 from Sample 1 and 736 from Sample 2. (cf. Figure 1)

Data were collected from late 2018 to early 2021 via an online questionnaire distributed on social media and through the university library system. Prior to completing the questionnaire, participants were informed about the purpose of the study, and provided written informed consent.

#### Questionnaire description

A version of HLS-EU-BR-Q47 was developed specifically for this survey that retained the dimensions and items of the HLS-EU-Q86 protocol (see Appendix A). Following the granting of permission to translate the HLS-EU questionnaire by the coordination of the European HLS-EU Consortium, the translation and back-translation processes ensured the initial phases of cultural adaptation of HLS-EU [47]. A team of three researchers with diverse backgrounds in linguistics, sociology, and education participated in the translation and back translation. This ensured that the final version would be informed by advanced knowledge and cultural experience.

The HLS-EU-BR-Q47 is a 47-item instrument based on the original instrument (HLS-EU-Q47), which encompasses four dimensions of HL: finding, understanding, appraising, and applying health information across three different domains: health care (16 items), disease prevention (16 items) and health promotion (15 items) [48]. Each item represents an activity associated with a specific dimension and domain. The respondents are requested to indicate their perceived competence to perform the specific activity on a four-point Likert scale with 1 representing “very easy”, 2 representing “easy”, 3 representing “difficult”, 4 representing “very difficult”. Consequently, we measured the self-perceived difficulty of selected health tasks. In order to facilitate interpretation, the values of each question were reversed so that higher scores indicated better HL. An overall HL score (overall index or general HL) and domain scores were calculated for each participant. Each score was subsequently standardized on a scale from 0 to 50, in accordance with the recommendations of the instrument’s developers [48].

Regular score calculation was obtained with the formula.

$$Index = (mean - 1)*\left( {\frac{{50}}{3}} \right)$$

The scores for general HL, health care HL, disease prevention HL, and health promotion HL were categorized into four levels: “inadequate HL” (score 0–25), “problematic HL” (score 25.01–33), “sufficient HL” (score 33.01–42), and “excellent HL” (score 42.01–50) [48]. The first two categories on this scale combined, collectively represents individuals with limited HL.

### Data analysis and statistics

Data analysis entailed the application of descriptive statistics, frequency tables, principal component analysis, and IRT. Pearson correlation analysis was conducted to examine the relationship between the IRT and HL scores. The statistical analyses were conducted using the software R (R Core Team, 2021) package MIRT (Chalmers, 2012).

#### Score reliability

In order to ascertain the instrument’s reliability, two distinct methodologies for measuring internal consistency were employed for comparative purposes: Cronbach’s Alpha [49] and McDonald’s Omega [50]. While Cronbach’s alpha is a reliability coefficient under the condition of one-dimensionality and tau equivalency. In contrast McDonald’s Omega is a congeneric reliability coefficient. The threshold values of the coefficients are 0.70 and 0.80, respectively [51].

#### Dimensionality

A full Information Factor Analysis (FIFA) was conducted based on the IRT methodology to validate the instrument’s dimensionality, specifically to ascertain the number of underlying factors involved, such as one-dimensionality of a factor. According to Reckase [52], the results may indicate the presence of a dominant factor if the first factor accounts for at least 20% of the total variance. The GRM is designed to evaluate a single latent trait. This type of analysis was selected for this study because it is more appropriate for a set of items with ordinal response categories [53], such as the HLS-EU-BR-Q47.

#### Model estimation - Samejima´s graded response model

In the Samejima-GRM [42–44], it is assumed that the response categories of an item are ordered among themselves. Suppose the categories of an item i are ordered from the lowest to the highest and are denoted by *k*_*i*_*= 0,1,2, …, m*_*i*_, where (*m*_*i*_ +1) is the number of categories of i-th. The probability that a respondent j chooses a particular or higher category of item i can be derived by extending the 2-parameter logistic model with the Eq. (1):

1 $$P^{+}_{i,{k}_{i}}\left({\theta }_{j}\right)=\frac{1}{1+{e}^{-{a}_{i}\left({\theta }_{j}-{b}_{i,ki}\right)}}$$,

with,

*i* = 1, 2, 3, …, *p; j* = 1, 2, 3, …, *n; k*_*i*_ = 0, 1, 2, …, *m*_*i*_),

where:

▪ *b*_*i, ki*_, is the difficulty parameter of the *k-*th category of item *i*. This parameter is known as the *threshold* parameter or location parameter. The difficulty parameter refers to the latent trait level, the probability that a respondent will select a response category k or a higher-ordered category is 0.50, with ${b}_{i,1}\le {b}_{i,2}\le \text{\ldots}\le {b}_{i,{m}_{i}}$;

▪ *a*_*i*_, is the item *i* discrimination parameter: this parameter represents the extent to which an item discriminates between respondents at different levels of the latent trait, determining the “quality” of the item. The greater the value of the parameter’s value, the better the item and the discrimination between respondents at different levels of the latent trait.

▪ *θ*_j_, the parameter of respondent *j* represents the respondent’s score, which is the respondent’s HL level within the IRT.

▪ ${P}_{i,ki}^{+\left({\theta }_{j}\right)}$ is the probability of the *j-th* respondent with an HL level of *θ*_j_ being in a particular category *k*_*i*_ or higher than the i-th level of the HL level, with ${P}^{+}_{i,0}\left({\theta }_{j}\right) =1$.

The probability of a respondent *j* to choose a category *k* on item *i* is given by Eq. (2) [42–44].

$$\eqalign{{P}_{i,{k}_{i}}\left({\theta }_{j}\right) &= P^{+}_{i,{k}_{i}}\left({\theta }_{j}\right)-P^{+}_{i,{k}_{i}+1}\left({\theta }_{j}\right) \cr & =\frac{1}{1+{e}^{-{a}_{i}\left({\theta }_{j}-{b}_{i,{k}_{i}}\right)}}-\frac{1}{1+{e}^{-{a}_{i}\left({\theta }_{j}-{b}_{i,{k}_{i}+1}\right)}}}$$

such that:

2 $${P^ + }_{i,0}({\theta _j}) = 1$$

It is observed that in an item with (*m*_*i*_*+ 1*) categories, *m*_*i*_ difficulty parameters need to be estimated in addition to the item’s discrimination parameter.

Consequently, the number of parameters to be estimated for each item is equal to the number of response categories minus 1. One of the contributions of IRT is the amount of information provided for each item at different levels of the latent trait scale. The information function indicates the region of the latent trait where an item best discriminates between respondents, that is, to what extent the item is better at each latent trait level. These functions can be calculated for each item. The information functions in IRT play a significant role in item description, as they guide the choice of items and also verify the efficiency between different items [39]. In order to determine the Item Information Function (FII) in the GRM, the following equation is employed:

3 $${I_i}(\theta ) = \sum\nolimits_{x = 1}^{{k_i}} {\frac{{{P^\prime}_{{\rm{ik}}}{{(\theta )}^2}}}{{{P_{{\rm{ik}}}}(\theta )}},}$$

where P_ik_’(θ) is the first derivative of the category response curve evaluated at a particular latent trait level. The total information curve of the test is the sum of all information functions of each item that composes it. The total information function is used to evaluate the performance of the items, that is, how well a set of items is evaluating the latent trait and is related to the precision needed to estimate the latent trait so that the standard error of measure can be estimated as the inverse of the square root of the total information value of the test at each level of the latent trait.

The GRM parameters can be estimated using Maximum Likelihood or Bayesian methods [54]. The estimation process of item and respondent parameters is implemented with software such as Multilog [55, 56], Parscale [56, 57], and MIRT package (multidimensional item response theory) [58] of R [59]. This research estimated the model´s items and the respondents’ parameters using the Maximum Marginal Likelihood and the EAP methods.

#### Estimating the HL level scale

Estimates of item parameters of the IRT model considered, i.e., *b*_*ik*_ (difficulty parameter), *a*_*i*_ (discrimination parameter), and HL level scores *θ*_*j*_ were obtained with the R software MIRT package [58]. The provided parameters’ estimates are on a scale (0, 1), where 0 represents the mean, and 1 is the standard deviation.

The HL scale was constructed using anchor levels and anchor items. For this construction, the cumulative probability was used to position the items on the scale according to their categories. Two consecutive levels in the latent trait are considered, X and Y, with X < Y an item *i* is measured as an anchor item at level Y [30], if:

i) a_i_ ≥1.
ii) P(U = 1|θ = Y) ≥ 0.60.

This way, the categories were positioned at the level where a respondent with this level has a probability of at least 0.60 to choose this category or higher. In addition, experts in the field propose cut-off points for the scale to improve its interpretation. In this study, the anchor levels were established and transformed on the scale [48, 10], where 50 represents the average HL level in the sample and 10 the standard deviation using the following Eqs. (4, 5, 6, and 7):

4 $${\rm{\theta }}* = 10 \times {\rm{ \theta }} + 50$$

5 $${\rm{b }}* = 10 \times {\rm{ b }} + 50$$

6 $${\rm{a }}* = {\rm{ a }}/10$$

7 $${\rm{P }}\left( {{\rm{Ui = 1 / \theta }}} \right){\rm{ = P }}\left( {{\rm{Ui = 1 / \theta *}}} \right)$$

where θ *, a *, and b * are the metric’s HL level score and item parameters [48, 10].

#### IRT and regular score correlations

A Pearson correlation analysis was conducted to assess whether there is a relationship between this HL regular score calculation and the IRT score calculation.

## Results

### Study sample

A total of1028 individuals participated in the study. The majority of participants were women (80.4%). Additionally, the 40–49 age group (58.1%) was the most represented in this study, compared to the other four age groups. The sample comprises 77.9% graduates from higher education, indicating a high level of education. Individuals from 26 provinces participated, with varying levels of participation, from 0.1% in Goías to 39.3% in Paraná (cf. Table 1).

**Table 1:** Statistics and demographic information about the survey respondents

	Class	#	(%)
Sex	Female		80.4
	Male		19.6
Age	18 to 29 years		13.9
	30 to 49 years		58.1
	50 to 59 years		19.6
	60 years or more		8.4
Educational Level	Without Completed Education Level		0.5
	Level 1 Complete Fundamental Ed		2.0
	Level 2 s stage of Fundamental E		1.5
	Level 3 Complete Secondary School		9.1
	Level 4 Technical training		8.1
	Level 5 University		25.3
	Level 6 Post-graduation lato senso		34.2
	Level 7 Post-graduation stricto senso		18.4
	Did not answer		0.9
Residence	00) No answer		1.5
	02) Alagoas		0.7
	04) Amazonas		0.3
	05) Bahia		0.04
	06) Ceará		3.4
	07) Distrito Federal		5.4
	08) Espírito Santo		7.6
	09) Goiás		0.1
	10) Maranhão		2.0
	12) Mato Grosso do Sul		0.5
	13) Minas Gerais		0.6
	14) Paraná		39.3
	15) Paraíba		3.8
	16) Pará		0.5
	17) Pernambuco		1.7
	18) Piauí		2.6
	19) Rio Grande do Norte		0.3
	20) Rio Grande do Sul		12.0
	21) Rio de Janeiro		6.5
	22) Rondônia		0.2
	23) Roraima		0.6
	24) Santa Catarina		3.6
	25) Sergipe		0.1
	26) São Paulo		6.4

### Data analysis and statistics

#### Instrument reliability

To assess the instrument’s reliability, Cronbach’s alpha and McDonald’s omega estimators of internal consistency were employed. The values of Cronbach’s alpha and McDonald’s omega were 0.96 and 0.97, respectively, indicating a very high level of internal consistency.

#### Dimensionality

The results of the explanatory factor analysis, based on IRT, indicated that the polychoric correlation matrix provides insights into the instrument dimensions, as illustrated in Fig. 1. In Appendix B, a table containing the eigenvalues is provided for reference.

The results show that the first component explains 51% (24 out of 47 items) of the total explained variance, indicating a dominant factor that meets Reckase’s [52] criterion for using a one-dimensional model of IRT.

The majority of items exhibited factor loadings exceeding 0.6 and communality values exceeding 0.3 in the exploratory factor analysis. Only the items HLHC01, HLHP34, HLHP45, and HLHP47 exhibited factor loadings between 0.5 and 0.6 and communality values below 0.3. Nevertheless, these items were retained in accordance with the recommendations set forth in [49], which stipulate that items with factor loadings exceeding 0.5 are deemed to be practically significant and relevant to the latent trait. Please refer to Appendix C for a table displaying the factor loadings and communality.

#### Model estimation - samejima ´s graded response model

The estimates of the item parameters on the scale (0, 1) are displayed in Table 2.

**Table 2:** Estimation of the item parameters and their respective standard errors (SE)

Item	a	SE	b1	SE	b2	SE	b3	SE
HLHC01	1.41	0.10	-2.97	0.21	-1.82	0.12	0.31	0.07
HLHC02	1.43	0.09	-2.84	0.19	-1.48	0.10	0.64	0.07
HLHC03	1.36	0.09	-2.58	0.17	-0.84	0.07	1.28	0.10
HLHC04	1.88	0.11	-2.36	0.14	-1.15	0.07	0.54	0.06
HLHC05	2.28	0.13	-2.26	0.13	-1.20	0.07	0.52	0.06
HLHC06	1.77	0.10	-2.04	0.12	-0.83	0.06	0.96	0.07
HLHC07	1.56	0.09	-2.18	0.13	-0.47	0.06	1.34	0.09
HLHC08	2.57	0.16	-2.46	0.15	-1.58	0.08	0.13	0.05
HLHC09	2.18	0.13	-2.46	0.15	-1.13	0.07	0.69	0.06
HLHC10	2.06	0.11	-2.07	0.11	-0.58	0.05	1.05	0.07
HLHC11	1.92	0.11	-2.11	0.12	-0.63	0.06	1.00	0.07
HLHC12	1.97	0.11	-1.76	0.10	-0.43	0.05	1.04	0.07
HLHC13	2.00	0.12	-2.59	0.16	-0.94	0.06	0.97	0.07
HLHC14	2.28	0.14	-2.60	0.16	-1.75	0.09	0.39	0.05
HLHC15	1.58	0.10	-2.63	0.17	-1.39	0.09	0.61	0.07
HLHC16	2.61	0.16	-2.48	0.15	-1.65	0.08	0.34	0.05
HLDP17	2.25	0.14	-2.58	0.16	-1.49	0.08	0.26	0.05
HLDP18	2.23	0.12	-2.12	0.12	-0.80	0.05	0.77	0.06
HLDP19	2.66	0.15	-2.33	0.13	-1.08	0.06	0.58	0.05
HLDP20	3.06	0.17	-2.14	0.11	-1.13	0.06	0.48	0.05
HLDP21	2.93	0.17	-2.34	0.13	-1.47	0.07	0.25	0.05
HLDP22	2.82	0.18	-2.52	0.16	-1.70	0.09	-0.15	0.04
HLDP23	3.30	0.21	-2.29	0.13	-1.62	0.08	-0.13	0.04
HLDP24	3.17	0.19	-2.23	0.12	-1.37	0.07	0.03	0.04
HLDP25	2.69	0.15	-2.11	0.11	-1.07	0.06	0.35	0.05
HLDP26	2.00	0.12	-2.48	0.15	-0.94	0.06	0.76	0.06
HLDP27	2.53	0.14	-2.29	0.13	-1.04	0.06	0.61	0.06
HLDP28	2.25	0.12	-2.11	0.12	-0.59	0.05	0.87	0.06
HLDP29	2.12	0.13	-2.63	0.17	-1.54	0.09	0.01	0.05
HLDP30	1.51	0.09	-2.50	0.16	-0.82	0.07	0.99	0.08
HLDP31	1.96	0.11	-2.18	0.13	-0.82	0.06	0.94	0.07
HLHP32	2.93	0.17	-2.44	0.15	-1.47	0.07	0.22	0.05
HLHP33	2.77	0.16	-2.37	0.14	-1.33	0.07	0.34	0.05
HLHP34	1.18	0.08	-1.89	0.13	0.15	0.07	1.82	0.13
HLHP35	1.45	0.09	-1.67	0.11	0.01	0.06	1.72	0.11
HLHP36	1.67	0.10	-2.00	0.12	-0.44	0.06	1.37	0.09
HLHP37	1.90	0.11	-2.37	0.14	-0.98	0.06	1.03	0.07
HLHP38	1.74	0.10	-1.99	0.12	-0.56	0.06	1.12	0.08
HLHP39	2.40	0.14	-2.33	0.13	-1.27	0.07	0.62	0.06
HLHP40	2.89	0.17	-2.38	0.14	-1.17	0.06	0.58	0.05
HLHP41	1.97	0.11	-2.34	0.14	-0.95	0.06	0.91	0.07
HLHP42	2.47	0.14	-2.37	0.14	-1.19	0.06	0.61	0.06
HLHP43	2.39	0.14	-2.75	0.18	-1.45	0.08	0.40	0.05
HLHP44	1.46	0.09	-2.73	0.18	-0.89	0.07	1.12	0.09
HLHP45	0.92	0.07	-2.28	0.19	-0.23	0.08	2.14	0.18
HLHP46	1.53	0.10	-2.63	0.17	-0.97	0.07	1.11	0.09
HLHP47	1.12	0.08	-2.59	0.19	-0.23	0.07	1.90	0.14

Note(s): a: discrimination parameter

b: difficulty parameter or location

(b_1_ = difficult, b_2_ = easy and b_3_ = very easy)

SE: standard error of the parameter’s estimates

Table 2 illustrates that the discrimination parameters ranged from 0.92 to 3.30 (a > 1), indicating that most items exhibited a high power of discrimination. Item 45, “being a member of a club, playing sports or taking a gym class”, exhibited the lowest value (a < 1), yet still demonstrated satisfactory power of discrimination. Item 23, “understand why you need health checks? (e.g., breast exam, blood sugar test, blood pressure),” exhibited the highest value.

Upon examination of the difficulty parameters, it becomes evident that b_1_ (“difficult”-response category) exhibited a range of -2.734 to -1.549, b_2_ (“easy”-response category) exhibited a range of -1.676 to 0.034, and b_3_ (“very easy”-response category) exhibited a range of -0.183 to 1.712. The b parameters are related to the latent trait level in which the probability of the respondent selecting a response category or a higher-ordered category is 0.50, so the parameter b_0_ (“very difficult”-response category) does not require estimation.

**Fig. 2:**
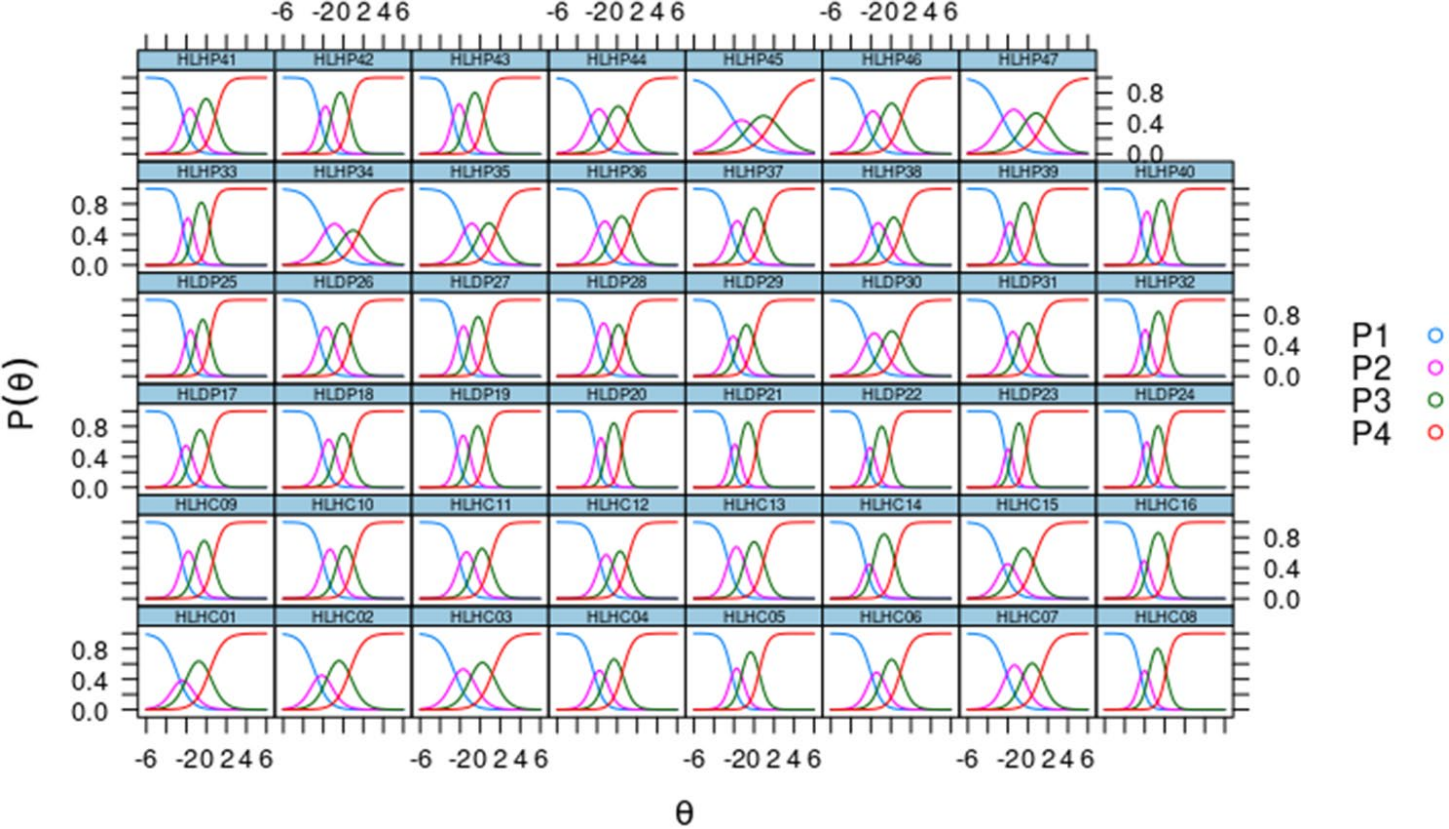
Graphs of the Characteristic Curve of the 47 items

Figure 2 depicts the Item Characteristic Curve (ICC) for the 47 items. This figure presents the categories on the graph’s curves, from left (P1 - very difficult) to right (P4 - very easy). However, most items have a region (interval) on the scale where each response category (P1, P2, P3, or P4) stands out. Some items’ intervals are small, indicating a lack of information for the category since it contains few responses. Although items 01, 08, 14, 16, 22, and 23 demonstrate satisfactory discrimination, their difficult category, P2, is a relatively narrow region that is not readily discernible. The remaining items are satisfactory in that they present all the curves of the categories in a manner that allows them to stand out in the region of the latent trait.

**Fig. 3:**
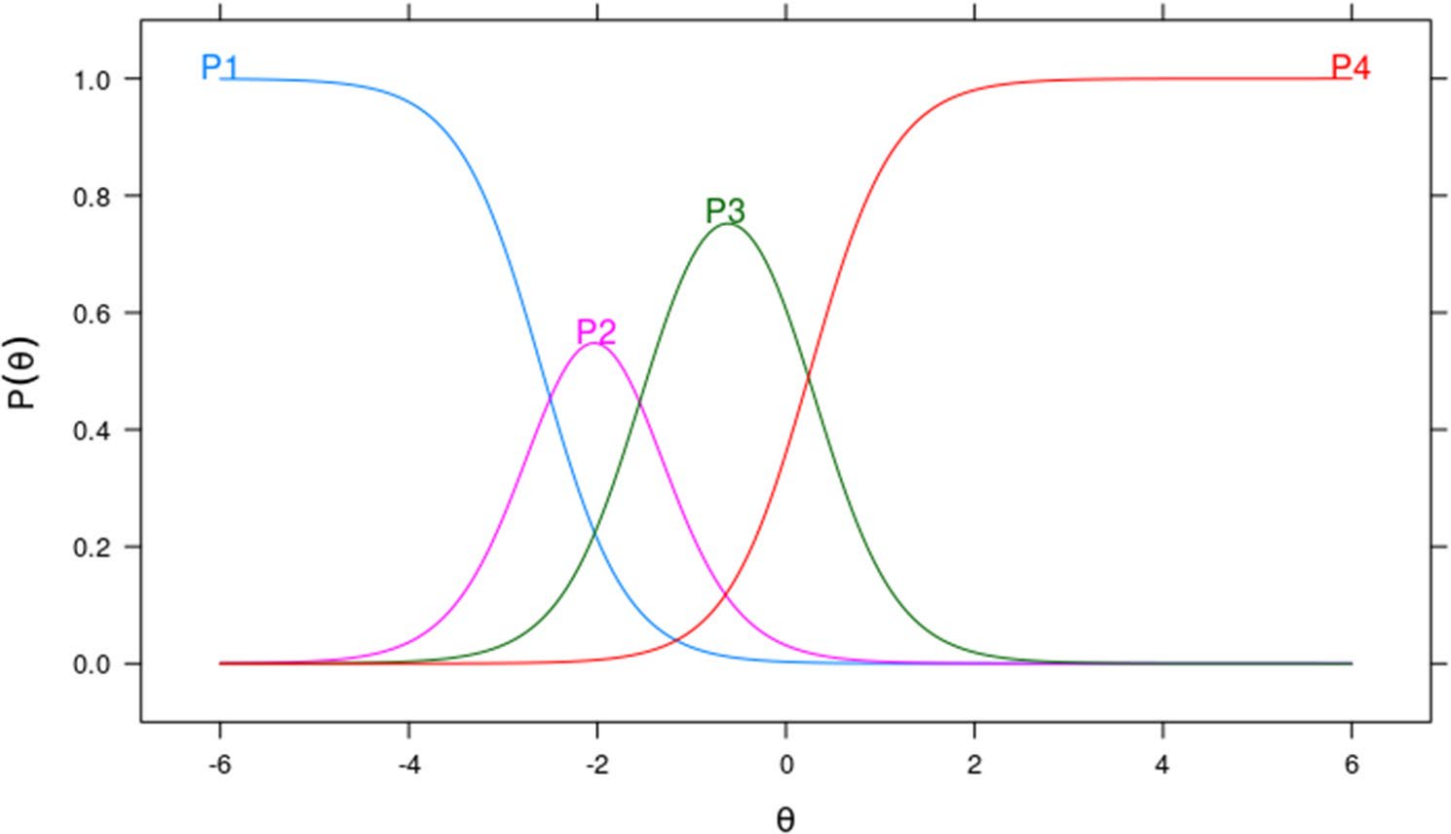
Graph of the Characteristic Curve of Item 17

Clarifying this methodology with a specific example of the Item Characteristic Curve (ICC), Fig. 3 shows the CCI for item 17 (“Finding information to manage behaviors that affect your health, such as smoking, insufficient physical activity and drinking too much alcohol”) with the following parameters: a = 2.25; b1 = -2.58; b2 = -1.49; b3 = 0.26. From this figure, the following interpretation emerges: Those respondents with latent trait values below approximately − 2.6 are more likely to respond to category 0 (P1 - very difficult). Those respondents with latent trait values approximately between − 2.6 and − 1.5 are more likely to respond to category 1 (P2 - difficult). Those respondents with latent trait values approximately between − 1.5 and 0.3 are more likely to respond to category 2 (P3 - easy). Finally, those respondents with latent trait values greater than 0.3 are more likely to respond to category 3 (P4 – very easy). The same interpretation can be applied to each of the remaining items.

**Fig. 4:**
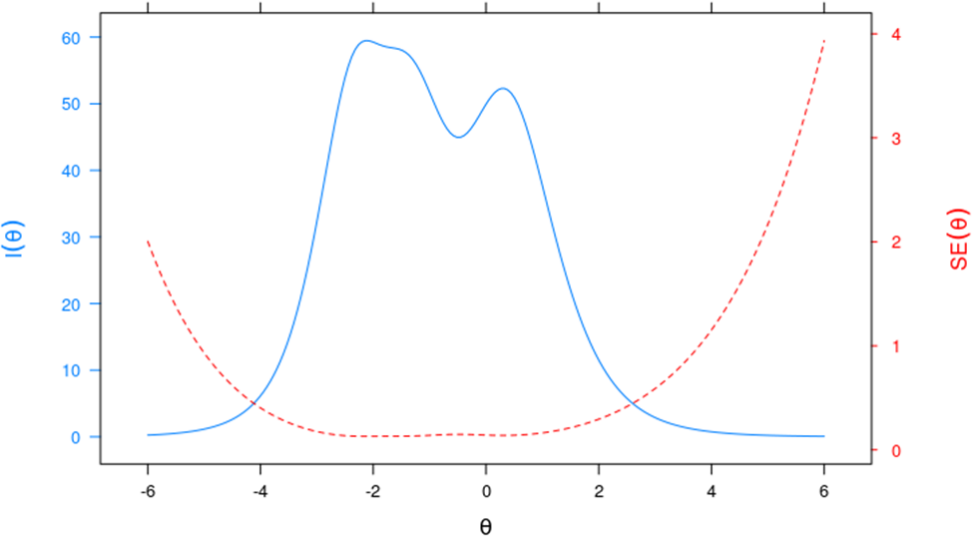
Test Information Function

Figure 4 depicts the Total Information Function (TIF) of the measurement instrument. It can be observed that the measurement instrument has a greater quantity of information, approximately in the range between − 4 and 2.3, which is consistent with the position of the items on the scale. In this interval, the standard error values are relatively low. Consequently, this questionnaire is more appropriate for measuring respondents with a HL level between − 4 and 2.3, i.e., from very low to high levels.

#### Estimating the HL level scale (based on IRT)

Following the estimation of the item parameters, the anchor categories and anchor levels were defined based on the conditions previously outlined before in Sect. 2.2.4 (Items 1 and 2) and transformed to a scale [48, 10], where 50 represents the average HL level in the sample and 10 the standard deviation where θ *, a *, and b * are the HL level score and the item parameters in the metric [48, 10].

**Fig. 5:**
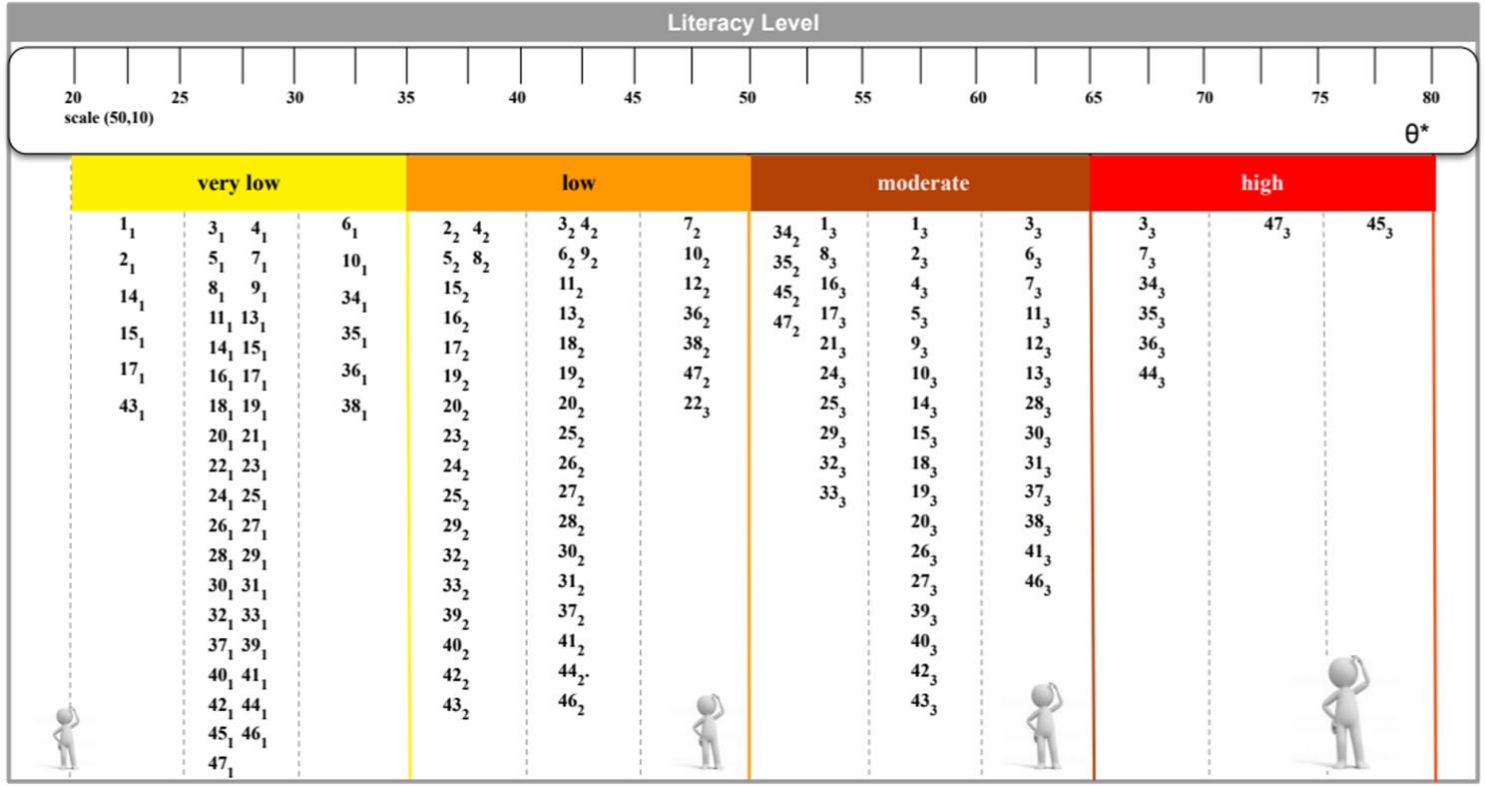
HL Level Scale

Figure 5 illustrates the anchor categories of the individual anchor levels (levels of the HL scale) on which the items were positioned. The respondents positioned below the lowest anchor level [35] rated all items as very difficult. Although Item 45 exhibited a discrimination parameter below 1, it was retained on the scale due to its thematic importance.

The interpretation of a participant’s position on the scale is based on his/her score, which indicates his/her level. The subsequent section will provide a detailed description of each HL level.

Very low level of HL (θ * ≤ 35) is defined as follows: This level is defined by identifying respondents who exhibited difficulty in answering the questions. This result is related to individuals who consider it very difficult and difficult to find information about symptoms and treatments of diseases that concern or cause concern, as well as find information about healthy activities, such as physical activity, healthy eating, and nutrition (1 to 2; 32). They also know what to do in the case of a medical emergency and get specialized help when they are sick, as well as understand the medical guidelines by following them and understand the package inserts of the medicines necessary for your treatment (3 to 6;11;14). The level of understanding still permeates difficulties about what to do in a medical emergency, in terms of how to ingest a prescribed medication and in the way of evaluating the advantages and disadvantages of different options in order to decide about their illness (7 to 10;13;15;16).

The respondents indicated that it was difficult or very difficult for them to find information about behaviors that affect their health, such as smoking, insufficient physical activity, and drinking too much alcohol. They also reported difficulty in finding information about managing mental health issues, stress, or depression; and in finding information, understanding and evaluating about vaccines and the reasons for taking them, as well as the need to understand and evaluate the relevance of the health exams that should be carried out as a way of preventing and controlling diseases, about health risks in the media are reliable (17 to 28; 43; 46).

The respondents indicated that it is difficult or very difficult to decide which vaccines to take, how to protect themselves from disease by specific sources (29 to 31; 39); how to gain more knowledge about well-being such as: meditation, walks, and Pilates; and to find information on how the neighborhood could be more health-friendly and activities that improve health and well-being in their community [33, 34, 41, 60, 42, 43, 45]. The respondents indicated that it is very difficult and difficult to know more about political changes that may affect health, and other information such as understanding the information on food packages (35 to 38); to assess how your housing compromises your health [61].

Low level of HL (35 < θ * ≤ 50) is indicated by: In addition to the aforementioned characteristics, this level is defined by identifying respondents who perceive it as easy to find information about treatments for diseases that concern them or cause concern, who find it easy to find out what to do in case of a medical emergency. This level is characterized by identifying respondents who consider it easy to find out what to do in a medical emergency and where to get specialized help when sick, such as from a doctor, pharmacist, or psychologist) (2 to 6). Furthermore, respondents find it easy to read medication inserts, follow medical guidelines, and evaluate treatment options. (7;10;11;13;15;16) At this level, respondents consider it easy to find information that interferes with physical and mental health, as well as what vaccines they may need, preventive exams and information about health risks in the media (17 to 20; 23 to 28; 43; 46). Respondents, at this level yet, consider it easy to decide which vaccines they need and want to take, how to protect themselves from the disease based on information from the media (29 to 31; 39); how to know more about well-being such as: meditation, walks, Pilates, among other healthy activities that their community offers. (33;40;41;44,47). At this level, the respondents consider it easy to learn more about efforts to promote their health at work; understand health advice from family and friends (36 to 38) and assess how their housing compromises their health (42). Furthermore, respondents at this level find it very easy to understand why they need vaccines (22).

Moderate level of HL (50 < θ * ≤ 65): This level is characterized by respondents who consider it easy to find information about how their neighborhood could be more health-friendly (e.g. reducing noise and pollution, to learn about creating green spaces, leisure), about policy changes that may affect health, e.g., legislation, health screening programs, new changes in government, restructuring of health services, among others (34 and 35). At this level, respondents perceive it easy to be a club member, practice sports or exercise classes, and get involved in activities that improve health and well-being in their community (45;47). Also, respondents perceive it very easy to find information regarding the symptoms and treatments of diseases that concern or cause concern, as well as to find information about healthy activities such as physical and mental activity and healthy eating (including understanding information presented on food packaging). Moreover, respondents find it very easy to understand instructions from their doctor or pharmacist on how to take a prescription drug, find it very easy to find information about treatments for diseases that concern them or cause concern, the leaflet that comes with the medication, to understand what to do in a medical emergency and to understand the information about physical and mental health that comes from the media (1; 2; 7 to 11;13 to 16; 31 to 33; 38 to 40). Furthermore, they consider it very easy to find information to manage your physical and mental health and about vaccinations and health checks you should have in order to prevent and control conditions such as overweight, high blood pressure or high cholesterol, as well as decide how you can protect yourself from the disease based on the advice of family and friends. (17 to 21; 24 to 30; and 37). At this level, respondents find it very easy to assess how the place where they live affects their health and well-being; how their housing conditions and decision-making for better daily behaviors (such as eating, physical and mental exercises, among others) contribute to maintaining a healthy life (41 to 44; 46).

High level of HL (θ * > 65). In addition to the characteristics of the previous items, respondents on this level find it very easy to find out what to do in a medical emergency, understand what to do in a medical emergency, learn more about political changes that can affect health, including health legislation, health screening programs, new governmental changes, and the education of health services (3; 7; 35). At this level, respondents perceive it very easy to find information that indicates how the neighborhood in which they live could be more health-friendly including the creation of spaces for leisure and enjoyment of green areas, learn how to promote health at work; to participate in activities designed to improve the health and well-being of the community and to make decisions that would improve their own health (34;36;44;45;47).

**Fig. 6:**
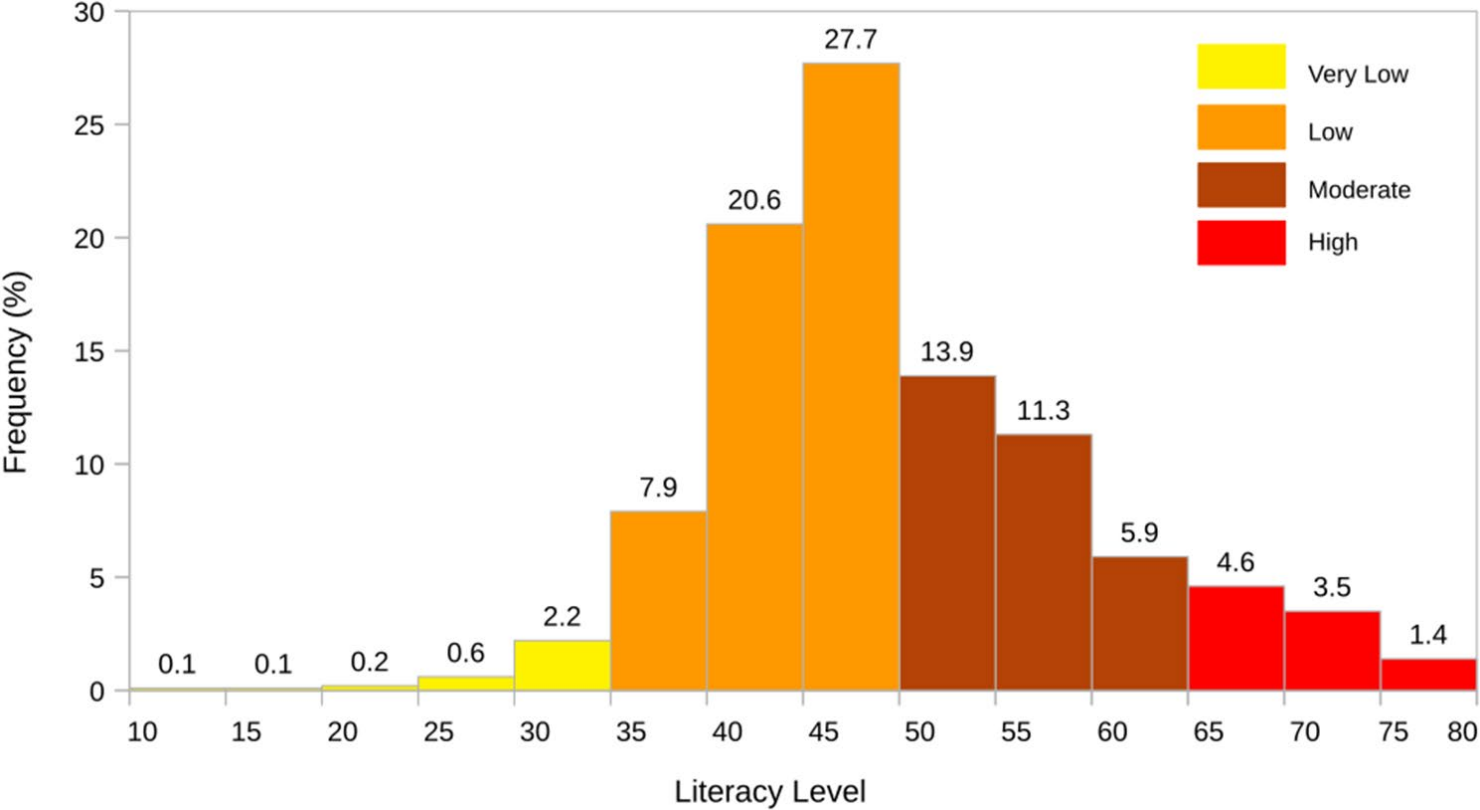
Distribution of respondents in the HL scale levels

Figure 6 depicts the histogram of the distribution of participants’ levels of HL. The majority of respondents (56.2%) exhibited values in the low level (35 < θ ≤ 50). A total of 3.2% of respondents exhibited a very low level of HL (θ < 35), 9.5% demonstrated a high level of HL, (65 ≤ θ) and 31.1% exhibited a moderate level of HL.

#### Levels of HL based on standard calculations

**Table 3:** Level of health literacy

		Frequency	Percent	Valid Percent	Cumulative Percent
Valid	Inadequate HL	109	10.4	10.8	10.8
	Problematic HL	399	37.9	39.5	50.2
	Sufficient HL	304	28.9	30.1	80.3
	Excellent HL	199	18.9	19.7	100.0
	Total	1011	96.1	100.0	
Missing	System	41	3.9		
Total		1052	100		

Table 3 displays the distribution of the HL levels based on the standard level computation. The mean HL scores were comparable between women and men (mean HLS-EU-Q: 33.9 vs. 33.7, *P* = 0.36). A significant association was observed between higher HL scores and younger age and higher educational and economic levels.

#### RT and regular score correlations

**Fig. 7:**
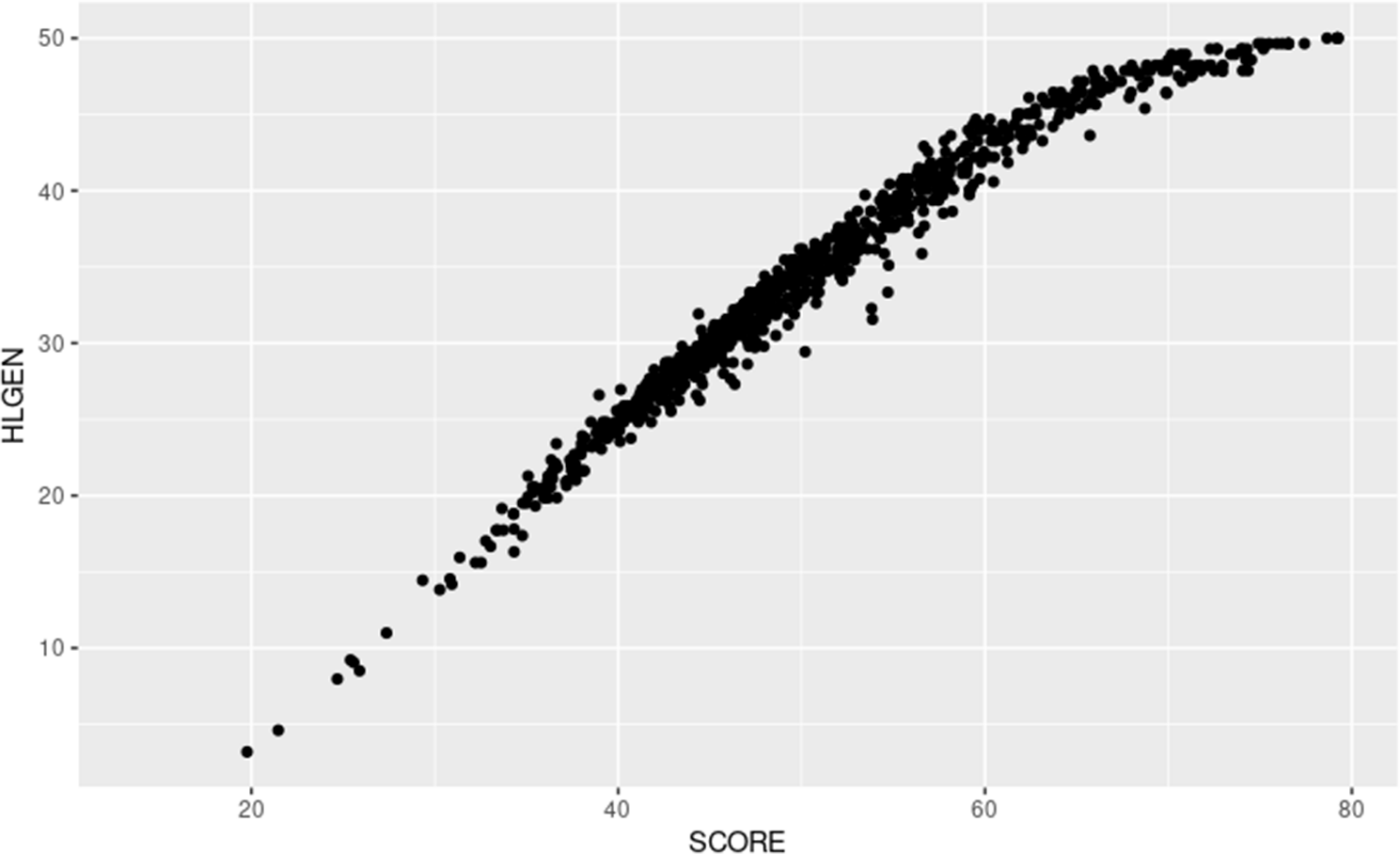
Scatter plot: HLGEN x SCORE

A correlation analysis was conducted to assess the relationship between the IRT and the literacy scores proposed for the standard calculation (cf. Figure 7). The scatter plot illustrates a discrepancy in the distribution of extreme values between the HLGEN measure and the values estimated by the IRT model. Nevertheless,, the scores are generally well estimated for the entire data distribution.

A high correlation was found, with the value of Pearson’s linear correlation coefficient equaling 0.9782.

## Discussion of results

This study aimed to provide evidence on the HL levels in Brazil using the HLS-EU-BR-Q47 survey. This evidence was gathered to provide evidence to support the construct validity (e.g., internal structure and invariance measurement) and reliability (score reliability) of the 47-item Health Literacy Survey using the Item Response Theory model.

### Limitations: sample and data collection procedures

This research endeavored to gather information from various regions within Brazil. The objective was not to achieve full representativeness, but rather to achieve saturation of the results in order to create a data set that could support the analysis of the implementation of IRT. Consequently, the data collection process, (online data collection using social media and university libraries, which includes a bias due to the self-selection process of the participants) served this purpose and was not a limitation. The data collected from almost all Brazilian states and diverse locations were normalized with an intentional bias according to educational level. (For example, given that HL is influenced by education level, selecting a set of participants who have a higher level of education than the average population. In our sample, 77.9% of the participants had a high level of education. This resulted in a sample that was likely to have a higher level of HL than the average Brazilian citizen [62]). Nevertheless, the majority of participants exhibited low levels of HL based on the statistical analysis. This suggests the need to investigate whether HL may be even of lower at the population level to capture the social hindrance for Brazilian citizens with low HL levels.

### Psychometric analysis of the tool – compared to other studies

The results of the IRT analysis unveil that the Brazilian Portuguese version of the HLS-EU-BR-Q47 questionnaire exhibited a one-dimensional characteristic. Furthermore, the Cronbach’s Alpha and Macdonald’s Omega coefficients demonstrated satisfactory levels of reliability and justify its use as a survey instrument to assess the level of HL in different populations, including in Brazil [19]. The Cronbach’s alpha and McDonald’s omega values of 0.96 and 0.97, respectively, indicate excellent accuracy [49, 63], thereby enabling the HL test to score participants with desired precision across the full range of regional and socioeconomic differences. Compared to other studies on the reliability of the HLS-EU-Q, which have reported Cronbach’s Alpha values between 0.51 and 0.91 [19], 0.84 in Romania [27] and 0.98 in Hindi [64], or 0.99 in Afghanistan [20], our study demonstrated a high level of accuracy.

The analysis of item loadings on factors revealed no interpretable pattern. The implications of this finding for the interpretation of this one-dimensional construct [39], could result from the similarity of the cognitive processes involved in response attributes or due to the closer relationship between the content domains for these items (even some overlapping of possible answers by participants). Consequently, these findings appear to align with a parsimonious approach, suggesting that a single, essential latent trait is being measured, namely general content knowledge in the context of HL.

The one-dimensional Samejima Gradual Response model proved adequate, as all items were well estimated. With the estimated parameters, it was possible to create the HL scale to cover all levels of HL, as indicated on the test information curve, since the items cover all levels of HL. An exploratory one-factor model fitted better than its two-factor counterpart or else (significant LRT test), at no cost for parsimony. The analysis of item loadings on factors revealed no interpretable pattern. This finding implies that the instrument can be assumed to be one-dimensional.

### HL levels

The IRT analysis of the HLS-EU-BR-Q47 data, allocated on a ruler, permitted the comparison between the items in relation to the difficulty or ease of answering the 47 questions in the three dimensions previously mentioned. This suggests that the instrument has an acceptable sensitivity. The *very low*, *low* and *moderate* HL levels were found in the three dimensions (health care, disease prevention and health promotion), and the high level was only identified in two dimensions (the health care and the health promotion dimension of HL). More participants were found to be less comfortable with questions related to health promotion and disease prevention than with questions related to disease management. These IRT results are consistent with the findings of other studies conducted in other parts of the world [26].

The finding that 56.2% of participants have a low HL level is consistent with findings from other studies employing the European HLS-EU instrument, which utilized classical HL computation: one in every two citizens had limited HL. However, the percentage of individuals with limited HL varied considerably by country, ranging from29%in the Netherlands to 62% in Bulgaria [7]. In Portugal, 59.9% and in a subsequent study, 49.0% of participants had limited general HL. Of these, 23.8% reported inadequate HL and 36.1% problematic HL [47]. The prevalence of low levels of HL among Brazilians was also evident in other Brazilian HL studies. These studies demonstrated that 31.7% or 32.4% (among the elderly even 51.6%) had an inadequate and marginal functional HL [23, 24].

### IRT and regular score correlations

IRT uses a different approach to access validity evidence; therefore, concurrent validity with other more traditional strategies was procured for this study. Through a correlation analysis between the scores of the HL level of each participant calculated with classical approaches and the scores obtained with IRT, a strong positive correlation was observed between the two methodologies which confer robust reliability in the use of the survey for research purposes.

Achieving quality of life, one output of a healthy lifestyle is based on society structural forms of organization. In this sense, the role of services and public policies, especially health policy and social participation become relevant determinants of health. Lack of access for disadvantaged groups makes it difficult to access health information, which is reflected in the answers on where to find relevant health information. By considering only individual issues like the items in the HLS-EU-BR-Q47 and not the context, it can often hide structural factors which affect HL in complex ways that do not depend on the citizen [65–67].

In order to help individuals at the very low and low levels of social strata improve their social condition, public policies focusing on HL improvement become crucial. Through skills and competences acquisition (with educational settings contribution, for example), information management (access, understand, apply) can contribute to increment knowledge used in decision-making favorable to health [2, 68].

## Conclusions

This cross-sectional study evaluated the psychometric properties of the European HL Survey Questionnaire translated into Portuguese (HLS-EU-BR-Q47) through the Item Response Theory (IRT) in a population-based sample of adults in Brazil. Using this HL instrument in cultural contexts other than the original one raises several issues, including the question of its validity. Consequently, we explored the psychometric properties of the HLS-EU-BR-Q47 based on data from most regions in Brazil.

The alarmingly low levels call for implementing more interventions to improve HL in Brazil.

These results contribute to the establishment of the instrument’s accuracy and provide favorable evidence for its use as a survey tool to measure the level of HL in varied populations.

The novelty of this study lies in the assessment of the accuracy of a HL instrument using two distinct statistical approaches: (Cronbach’s Alpha, Item Response Theory and the Item Characteristic Curve and HLGEN measure. As both approaches have given evidence for the scale’s accuracy, we invite other researchers to consider using various statistical approaches to verify their HL measures. Based on this validated instrument, we can now conclude that it is an appropriate instrument for assessing HL in Brazil. Moreover, this study provides novel knowledge and evidence on the validity of one of the HL instruments from the South American (Brazilian) population. This instrument can be used by citizens, health professionals, decision-makers, or regional/national policymakers to develop initiatives to access and increase the HL of individuals, groups, and communities. To confirm and expand the findings, future studies are needed to fill the gap not covered in this research on the influence of local cultures and practices in the vast Brazilian territory.

### Electronic supplementary material

Below is the link to the electronic supplementary material.

Supplementary Material 1

Supplementary Material 2

Supplementary Material 3

Supplementary Material 4

## Data Availability

The datasets used and/or analyzed during the current study available from the corresponding author on reasonable request.

## Abbreviations

a: Item discrimination parameters
ANOVA: Variance Analyze
bi,k: Item category parameters
BIB: Incomplete Balanced Blocs
CMT: Classic Measurement Theory
EP: Expected Posterior
FIT: Total Information Function of the Test
GRM: Gradual Response Model
HLS-EU-BR-Q47: Questionnaire used for the health literacy survey in Europe translated for Brazil including 47 questions
i: Item or indicator
ICC: Item Characteristic Curve
IIQ: Item Information Quantity
IRT: Item Response Theory
ki: Category of an item i
MP: Mode Posteriori
MV: Maximum Likelihood Method
OLS: Ordinary Least Square Regression
Pi,k: Probability of the j-th respondent, with the sustainability level

## References

[1] Nutbeam D (2000). Health literacy as a public health goal: a challenge for contemporary health education and communication strategies into the 21st century. Health Promot Int.

[2] Van den Sørensen K, Fullam J, Doyle G, Pelikan J, Slonska Z (2012). Health literacy and public health: a systematic review and integration of definitions and models. BMC Public Health.

[3] World Health Organization. Shanghai Declaration on promoting health in the 2030 Agenda for Sustainable Development. 2016. 10.1093/heapro/daw103 28180270

[4] World Health Organization. Geneva Charter for Well-being. 2022.

[5] World Health Organization. Ottawa Charter for Health Promotion. 1986.

[6] World Health Organization. Nairobi Call to Action. 2009.

[7] Sørensen K, Pelikan JM, Röthlin F, Ganahl K, Slonska Z, Doyle G (2015). Health literacy in Europe: comparative results of the European health literacy survey (HLS-EU). Eur J Public Health.

[8] Duong TV, Aringazina A, Baisunova G, Nurjanah, Pham TV, Pham KM (2017). Measuring health literacy in Asia: validation of the HLS-EU-Q47 survey tool in six Asian countries. J Epidemiol.

[9] Paasche-Orlow M. Health Literacy Tool Shed: A database of health literacy measures. 2021.

[10] Parker RM, Baker DW, Williams MV, Nurss JR (1995). The test of functional health literacy in adults. J Gen Intern Med.

[11] Davis TC, Crouch MA, Long SW, Jackson RH, Bates P, George RB (1991). Rapid assessment of literacy levels of adult primary care patients. Fam Med.

[12] Weiss BD, Mays MZ, Martz W, Castro KM, DeWalt DA, Pignone MP (2005). Quick Assessment of Literacy in primary care: the Newest Vital sign. Ann Fam Med.

[13] Berkman ND, Sheridan SL, Donahue KE, Halpern DJ, Viera A, Crotty K et al. Health literacy interventions and outcomes: an updated systematic review. Evid Rep Technol Assess Full Rep. 2011;199. PMC4781058 23126607

[14] Kutner M, Greenberg, Elizabeth, Jin Y. Paulsen C. The Health Literacy of America’s Adults Results From the 2003 National Assessment of Adult Literacy. 2006.

[15] Wang J, Thombs BD, Schmid MR (2014). The Swiss health literacy survey: development and psychometric properties of a multidimensional instrument to assess competencies for health. Health Expect.

[16] Tavousi M, Haeri-Mehrizi A, Rakhshani F, Rafiefar S, Soleymanian A, Sarbandi F (2020). Development and validation of a short and easy-to-use instrument for measuring health literacy: the health literacy instrument for adults (HELIA). BMC Public Health.

[17] Osborne RH, Batterham RW, Elsworth GR, Hawkins M, Buchbinder R (2013). The grounded psychometric development and initial validation of the health literacy questionnaire (HLQ). BMC Public Health.

[18] Tufts Medicine. Health Literacy Tool Shed. Health Literacy Tool Shed. https://healthliteracy.tuftsmedicine.org/. Accessed 14 May 2024.

[19] Van den Sørensen K, Pelikan JM, Fullam J, Doyle G, Slonska Z (2013). Measuring health literacy in populations: illuminating the design and development process of the European Health Literacy Survey Questionnaire (HLS-EU-Q). BMC Public Health.

[20] Harsch S, Jawid A, Jawid E, Saboga-Nunes L, Sørensen K, Sahrai D (2021). Health Literacy and Health behavior among women in Ghazni, Afghanistan. Front Public Health.

[21] de Castro SH, Brito GNO, Gomes MB (2014). Health literacy skills in type 2 diabetes mellitus outpatients from an university-affiliated hospital in Rio De Janeiro, Brazil. Diabetol Metab Syndr.

[22] Batista MJ, Lawrence HP, Sousa M (2017). Da LR de. Oral health literacy and oral health outcomes in an adult population in Brazil. BMC Public Health.

[23] Carthery-Goulart MT, Anghinah R, Areza-Fegyveres R, Bahia VS, Brucki SMD, Damin A (2009). Performance of a Brazilian population on the test of functional health literacy in adults. Rev Saúde Pública.

[24] Apolinario D, Mansur LL, Carthery-Goulart MT, Brucki SMD, Nitrini R (2014). Detecting limited health literacy in Brazil: development of a multidimensional screening tool. Health Promot Int.

[25] Moraes KL, Brasil VV, Mialhe FL, Sampaio HA, de Sousa C, Canhestro ALL. MR, Validation of the Health Literacy Questionnaire (HLQ) to Brazilian Portuguese. Acta Paul Enferm. 2021;34.

[26] Pelikan JM, Ganahl K (2017). Measuring Health Literacy in general populations: primary findings from the HLS-EU Consortium’s health literacy Assessment Effort. Stud Health Technol Inf.

[27] Coman MA, Van den Forray AI, Chereches RM (2022). Measuring Health Literacy in Romania: validation of the HLS-EU-Q16 Survey Questionnaire. Int J Public Health.

[28] CASTRO R, SANTOS T, TRIERWEILLER A, CAMPOS L. BORNIA A. Teoria da Resposta ao Item: Levantamento Exploratório da Produção Científica. 2013.

[29] Moreira Junior F, de Zanella J, Lopes A, Seidel LFD (2015). Avaliação Da satisfação De alunos por meio do Modelo De Resposta Gradual Da Teoria Da Resposta Ao Item. Ens Aval E Políticas Públicas. Em Educ.

[30] Schmitt J, Fini MI, Bailer C, Fritsch R, Andrade DF. de. WWH-dropout scale: when, why and how to measure propensity to drop out of undergraduate courses. J Appl Res High Educ. 2020;13:540–60.

[31] Rodrigues MTP, Moreira TMM, de Andrade DF (2014). Elaboração E validação de instrumento avaliador da adesão ao tratamento da hipertensão. Rev Saúde Pública.

[32] Menegon Lda, Vincenzi S, de Andrade SL, Barbetta DF, Merino PA, Vink EAD (2017). Design and validation of an aircraft seat comfort scale using item response theory. Appl Ergon.

[33] Menegon Lda, Vincenzi S, Andrade SL, de Barbetta DF, Vink PA, Merino P (2019). An aircraft seat discomfort scale using item response theory. Appl Ergon.

[34] Mâsse LC, O’Connor TM, Lin Y, Hughes SO, Tugault-Lafleur CN, Baranowski T (2020). Calibration of the food parenting practice (FPP) item bank: tools for improving the measurement of food parenting practices of parents of 5–12-year-old children. Int J Behav Nutr Phys Act.

[35] Santos TSS, Araújo PH, de Andrade M, de Louzada DF, Assis ML, de Slater MAA. B. Two validity evidences of the ESQUADA and Brazilians’ dietary quality levels. Rev Saúde Pública. 2021;55. 10.11606/s1518-8787.2021055002397 PMC8352565 34406276

[36] Tezza R, Bornia AC, de Andrade DF, Barbetta PA (2018). Multidimensional model to measure quality in e-commerce websites using item response theory. Gest Produção.

[37] de Araujo EAC, de Andrade DF, Bortolotti SLV (2009). Teoria Da Resposta Ao Item. Rev Esc Enferm USP.

[38] Pasquali L, Psicometria (2013). Teoria dos testes na psicologia e na educação. 5a edição.

[39] Hambleton RK, Swaminathan H, Rogers HJ (1991). Fundamentals of item response theory.

[40] de Andrade DF, Tavares HR. Teoria da Resposta ao Item: Conceitos e Aplica¸co˜es. SIMPÓSIO Nac PROBABILIDADE E Estat - SINAPE. 2000.

[41] Embretson SE, Reise SP. Item response theory. Psychology; 2013.

[42] Samejima F (1968). Estimation of latent ability using a response pattern of graded Scores1. ETS Res Bull Ser.

[43] Samejima F. A General Model for Free Response Data. Psychometrika. 1972.

[44] Samejima F, n der Linden WJ, Hambleton RK (1997). Graded response model. Handbook of modern item response theory.

[45] Field A, Discovering Statistics Using SPSS. 3rd Edition. 3rd Edition. Los Angeles: SAGE Publications Ltd; 2009.

[46] Hambleton RK. Principles and selected applications of item response theory. Educational measurement. 3rd ed. American Council on Education; 1989. pp. 147–200.

[47] Martins R, Nunes LS. The challenges of epistemological validation to Brazil of the European Health Literacy Survey (HLS-EU-BR): World Congress of Health Research. Aten Primaria. 2014;46 Espec Cong 1:12.

[48] The HLS-EU Consortium. Measurement of health literacy in Europe: HLS-EU-Q47; HLS-EU-Q16; and HLS-EU-Q86. 2012.

[49] Hair J. Multivariate Data Analysis. Fac Publ. 2009.

[50] McDonald RP (1999). A unified treatment.

[51] Nunnaly JC, Bernstein IH. Construction of conventional tests. Psychom Theory. 1994;:293–323.

[52] Reckase MD (1979). Unifactor Latent Trait models Applied to Multifactor tests: results and implications. J Educ Stat.

[53] Joreskog KG, Moustaki I. Factor Analysis of Ordinal Variables with Full Information Maximum Likelihood. 2006.

[54] Baker FB, Kim S-H, editors. Item Response Theory: Parameter Estimation Techniques, Second Edition. 2nd edition. Boca Raton: CRC Press; 2014.

[55] Thissen D. Multilog user’s guide: multiple, categorical item analysis and test Scoring using item response theory. Scientific Software International, Incorporated; 1991.

[56] Toit MD. IRT from SSI: BILOG-MG, MULTILOG, PARSCALE, TESTFACT. Scientific Software International; 2003.

[57] Muraki E, Bock RD, Parscale. IRT Based Test Scoring and Item Analysis for Graded Open-Ended exercises and performance tasks. Scientific Software International, Incorporated; 1993.

[58] Chalmers RP (2012). Mirt: a Multidimensional Item Response Theory Package for the R environment. J Stat Softw.

[59] R Core Team. R: A language and environment for statistical computing. 2021.

[60] de Fragoso T. M. Modelos multidimensionais da teoria de resposta ao item. PhD Thesis. Universidade de São Paulo; 2010.

[61] Tezza R. Modelagem multidimensional para mensurar qualidade em website de e-commerce utilizando a teoria da resposta ao item. Doutorado em Engenharia De Produção. Universidade Federal de Santa Catarina; 2012.

[62] OECD. Education at a glance 2022: OECD indicators. OECD; 2022.

[63] Viladrich C, Angulo-Brunet A, Doval E (2017). Un viaje alrededor de alfa y omega para estimar la fiabilidad de consistencia interna. Psicol Ann Psychol.

[64] Van den Dsouza JP, Pattanshetty S (2021). Validity and reliability of the Indian Version of the HLS-EU-Q16 Questionnaire. Int J Environ Res Public Health.

[65] Viswanath K, Ackerson LK (2011). Race, ethnicity, language, social class, and health communication inequalities: a nationally-representative cross-sectional study. PLoS ONE.

[66] McCloud RF, Jung M, Gray SW, Viswanath K (2013). Class, race and ethnicity and information avoidance among cancer survivors. Br J Cancer.

[67] Kelley MS, Su D, Britigan DH (2016). Disparities in Health Information Access: results of a county-wide survey and implications for Health Communication. Health Commun.

[68] Pina ALS. Literacia em saúde e o impacto sobre a gestão da saúde: comportamentos de saúde de estudantes de países africanos de língua oficial portuguesa. masterThesis. 2020.

