# Supplementary material for: Psychometric properties of the health literacy instrument in Brazil (HLS-EU-BR47)
Source: BMC Public Health. 2024 Jun 20;24:1655. doi: 10.1186/s12889-024-19108-2 (PMC11191178; doi:10.1186/s12889-024-19108-2)
Supplement: Supplementary file 1 — Supplementary Material 1 [file 12889_2024_19108_MOESM1_ESM.docx]

**Appendix B**

Table with eigenvalues

| **PC** | **Eigenvalues** | **% variance explicated** | **Eigenvalues accumulated** | **Percantage of variance accumulated** |
| --- | --- | --- | --- | --- |
| 1 | 24.34 | 51.80% | 24.34 | 51.80% |
| 2 | 2.81 | 5.98% | 27.16 | 57.78% |
| 3 | 2.09 | 4.45% | 29.25 | 62.22% |
| 4 | 1.42 | 3.01% | 30.66 | 65.24% |
| 5 | 1.36 | 2.89% | 32.02 | 68.13% |
| 6 | 1.11 | 2.36% | 33.13 | 70.49% |
| 7 | 0.99 | 2.10% | 34.12 | 72.59% |
| 8 | 0.94 | 2.00% | 35.06 | 74.60% |
| 9 | 0.80 | 1.71% | 35.86 | 76.31% |
| 10 | 0.73 | 1.56% | 36.60 | 77.86% |
| 11 | 0.67 | 1.42% | 37.26 | 79.29% |
| 12 | 0.61 | 1.30% | 37.88 | 80.59% |
| 13 | 0.58 | 1.23% | 38.45 | 81.82% |
| 14 | 0.56 | 1.19% | 39.01 | 83.00% |
| 15 | 0.51 | 1.08% | 39.52 | 84.09% |
| 16 | 0.49 | 1.04% | 40.01 | 85.13% |
| 17 | 0.47 | 0.99% | 40.48 | 86.12% |
| 18 | 0.45 | 0.95% | 40.92 | 87.07% |
| 19 | 0.42 | 0.89% | 41.34 | 87.96% |
| 20 | 0.40 | 0.85% | 41.74 | 88.80% |
| 21 | 0.39 | 0.82% | 42.12 | 89.62% |
| 22 | 0.36 | 0.76% | 42.48 | 90.38% |
| 23 | 0.35 | 0.74% | 42.83 | 91.12% |
| 24 | 0.31 | 0.66% | 43.14 | 91.78% |
| 25 | 0.30 | 0.65% | 43.44 | 92.43% |
| 26 | 0.29 | 0.62% | 43.73 | 93.05% |
| 27 | 0.27 | 0.58% | 44.00 | 93.62% |
| 28 | 0.25 | 0.54% | 44.26 | 94.16% |
| 29 | 0.24 | 0.52% | 44.50 | 94.68% |
| 30 | 0.22 | 0.47% | 44.72 | 95.15% |
| 31 | 0.21 | 0.46% | 44.94 | 95.61% |
| 32 | 0.21 | 0.44% | 45.14 | 96.05% |
| 33 | 0.20 | 0.42% | 45.34 | 96.47% |
| 34 | 0.18 | 0.38% | 45.52 | 96.85% |
| 35 | 0.17 | 0.36% | 45.69 | 97.21% |
| 36 | 0.16 | 0.33% | 45.85 | 97.55% |
| 37 | 0.15 | 0.33% | 46.00 | 97.87% |
| 38 | 0.14 | 0.30% | 46.14 | 98.18% |
| 39 | 0.14 | 0.29% | 46.28 | 98.47% |
| 40 | 0.12 | 0.25% | 46.40 | 98.71% |
| 41 | 0.11 | 0.24% | 46.51 | 98.95% |
| 42 | 0.11 | 0.23% | 46.61 | 99.18% |
| 43 | 0.10 | 0.20% | 46.71 | 99.38% |
| 44 | 0.08 | 0.18% | 46.79 | 99.56% |
| 45 | 0.08 | 0.17% | 46.87 | 99.73% |
| 46 | 0.07 | 0.14% | 46.94 | 99.87% |
| 47 | 0.06 | 0.13% | 47.00 | 100.00% |
