# Supplementary material for: Psychometric properties of the health literacy instrument in Brazil (HLS-EU-BR47)
Source: BMC Public Health. 2024 Jun 20;24:1655. doi: 10.1186/s12889-024-19108-2 (PMC11191178; doi:10.1186/s12889-024-19108-2)
Supplement: Supplementary file 2 — Supplementary Material 2 [file 12889_2024_19108_MOESM2_ESM.pdf]

APPENDIX A_Portuguese

1) Instrument introduction and informed consent

2) Health Literacy (HL) perception, measured with an instrument with 47 items described below (in Portuguese)

3) Demographic information

1) **Instrument introduction and informed consent**

LITERACIA PARA A SAÚDE: A VOZ DA COMUNIDADE NA REDE BIBLIOSUS

A pesquisa se propõe a responder à questão: Como as pessoas da comunidade expressam suas necessidades de informação e o nível de literacia para a saúde relacionadas à doença, à qualidade de vida e à promoção da saúde junto à Rede BiblioSUS?
Esta pesquisa constitui um estudo quali-quantitativo com aplicação do questionário on-line: Literacia para a Saúde (HLS-EU-BR).

Agradecemos a participação e colocando-nos à disposição pelo e-mail: capagiic@ufrgs.br.

Atenciosamente,
Grupo de Pesquisa LEIA e Rede Lusófona de Literacia para a Saúde (210507)

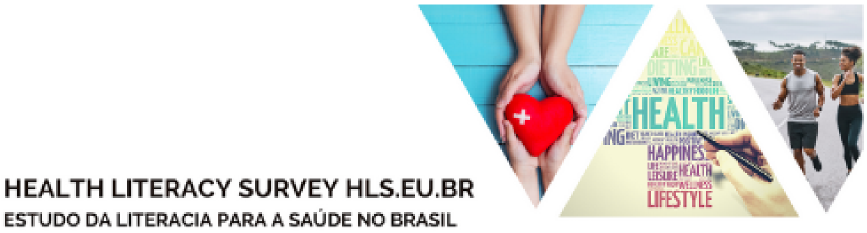

TERMO DE CONSENTIMENTO LIVRE E ESCLARECIDO (TCLE) PARA QUESTIONÁRIO ON-LINE *

Convidamos a participar voluntariamente no questionário on-line do Projeto de Pesquisa "Literacia para a Saúde: a voz da comunidade na Rede BiblioSUS", realizada pelo Grupo Interinstitucional de Pesquisa LEIA: Leitura, Informação e Acessibilidade e pela Rede Lusófona de Literacia para a Saúde. A pesquisa tem como pesquisador responsável Profª Drª Eliane Lourdes da Silva Moro, com coordenação adjunta da Profª Drª Lizandra Brasil Estabel e está cadastrada no CEP/UFRGS. O tempo estimado para responder a totalidade das questões é de, aproximadamente, 30 min. O estudo visa responder à questão: Como as pessoas da comunidade expressam suas necessidades de informação e o nível de literacia para a saúde relacionadas à doença, à qualidade de vida e à promoção da saúde junto à Rede BiblioSUS? Os objetivos consistem em identificar as necessidades de informação e o nível de literacia para a saúde relacionadas à doença, à qualidade de vida e à promoção da saúde da comunidade no acesso e uso da rede BiblioSUS e analisar a comunidade nas suas necessidades em relação às Tecnologias de Informação e de Comunicação (TIC). Os dados coletados pelo presente questionário on-line serão utilizados para fins acadêmicos, na parceria entre a Universidade Federal do Rio Grande do Sul (UFRGS) e o Ministério da Saúde, sendo preservada a sua confidencialidade e garantido o sigilo das informações recolhidas. Agradecemos as informações prestadas colocando-nos à disposição para quaisquer esclarecimentos.

- [ ] Concordo em participar da pesquisa
- [ ] Não concordo em participar da pesquisa

Question *

Não existem respostas certas ou erradas. Os resultados obtidos são confidenciais e utilizados para fins meramente estatísticos. Indique, por favor, a hora a que vai iniciar o preenchimento do questionário:

2) **Health Literacy (HL) perception, measured with an instrument with 47 items described below (in Portuguese)**

Numa escala que vai de muito fácil a muito difícil, quão fácil diria que é

1 muito fácil 2 fácil 3 difícil 4 muito difícil 5 não sabe/ não responde

1 "... encontrar informações sobre sintomas de doenças que lhe dizem respeito ou causam preocupação?"
2 "... encontrar informações sobre tratamentos de doenças que lhe dizem respeito ou causam preocupação?"
3 "... descobrir o que fazer em caso de uma emergência médica?"
4 "... descobrir onde obter ajuda especializada quando está doente? (por ex. junto de um médico, farmacêutico, psicólogo)"
5 "... compreender o que o seu médico lhe diz?"
6 "... compreender a bula (os folhetos) que acompanham o seu medicamento?"
7 "... compreender o que fazer numa emergência médica?"
8 "... compreender instruções do seu médico ou farmacêutico sobre o modo como tomar um medicamento receitado?"
9 "... avaliar como é que a informação oriunda do seu médico se aplica ao seu caso?"
10 "... avaliar vantagens e desvantagens de diferentes opções de tratamento?"
11 "... avaliar quando pode necessitar de uma segunda opinião de outro médico?"
12 "... avaliar se a informação sobre a doença nos meios de comunicação é de confiança?" (por ex. TV, internet ou outros meios de comunicação)
1 "... encontrar informações sobre sintomas de doenças que lhe dizem respeito ou causam preocupação?"
2 "... encontrar informações sobre tratamentos de doenças que lhe dizem respeito ou causam preocupação?"
3 "... descobrir o que fazer em caso de uma emergência médica?"
4 "... descobrir onde obter ajuda especializada quando está doente? (por ex. junto de um médico, farmacêutico, psicólogo)"
5 "... compreender o que o seu médico lhe diz?"
6 "... compreender a bula (os folhetos) que acompanham o seu medicamento?"
7 "... compreender o que fazer numa emergência médica?"
8 "... compreender instruções do seu médico ou farmacêutico sobre o modo como tomar um medicamento receitado?"
9 "... avaliar como é que a informação oriunda do seu médico se aplica ao seu caso?"
10 "... avaliar vantagens e desvantagens de diferentes opções de tratamento?"
11 "... avaliar quando pode necessitar de uma segunda opinião de outro médico?"
12 "... avaliar se a informação sobre a doença nos meios de comunicação é de confiança?" (por ex. TV, internet ou outros meios de comunicação)
13 "... usar informações que o seu médico lhe dá para tomar decisões sobre a sua doença?"
14 "... seguir instruções sobre medicação?"
15 "... chamar uma ambulância em caso de emergência?"
16 "... seguir as instruções do seu médico ou farmacêutico?"
17 "... encontrar informações para gerir comportamentos que afetam a sua saúde, tais como fumar, atividade física insuficiente e beber álcool em demasia?"
18 "... encontrar informações para gerir problemas de saúde mental, tais como stresse ou depressão?"
19 "... encontrar informações sobre vacinas e exames de saúde que devia fazer?" (por ex. exame de mama, teste de açúcar no sangue, pressão arterial)
20 "... encontrar informações sobre como prevenir ou controlar condições, tais como o excesso de peso, pressão arterial alta ou colesterol alto?"
21 "... compreender advertências relativas à saúde e comportamentos, tais como fumar, atividade física insuficiente e beber álcool em demasia?"
22 "... entender porque precisa de vacinas?"

23 "... entender porque precisa de exames de saúde? (por ex. exame de mama, teste de açúcar no sangue, pressão sanguínea)"
24 "... avaliar quão seguras são as advertências envolvendo a saúde, tais como fumar, atividade física insuficiente e beber álcool em demasia?"
25 "... avaliar quando precisa de ir a um médico para um check-up ou exame geral de saúde?"
13 "... usar informações que o seu médico lhe dá para tomar decisões sobre a sua doença?"
14 "... seguir instruções sobre medicação?"
15 "... chamar uma ambulância em caso de emergência?"
16 "... seguir as instruções do seu médico ou farmacêutico?"
17 "... encontrar informações para gerir comportamentos que afetam a sua saúde, tais como fumar, atividade física insuficiente e beber álcool em demasia?"
18 "... encontrar informações para gerir problemas de saúde mental, tais como stresse ou depressão?"
19 "... encontrar informações sobre vacinas e exames de saúde que devia fazer?" (por ex. exame de mama, teste de açúcar no sangue, pressão arterial)
20 "... encontrar informações sobre como prevenir ou controlar condições, tais como o excesso de peso, pressão arterial alta ou colesterol alto?"
21 "... compreender advertências relativas à saúde e comportamentos, tais como fumar, atividade física insuficiente e beber álcool em demasia?"
22 "... entender porque precisa de vacinas?"
23 "... entender porque precisa de exames de saúde? (por ex. exame de mama, teste de açúcar no sangue, pressão sanguínea)"
24 "... avaliar quão seguras são as advertências envolvendo a saúde, tais como fumar, atividade física insuficiente e beber álcool em demasia?"
25 "... avaliar quando precisa de ir a um médico para um check-up ou exame geral de saúde?"
26 "... avaliar quais são as vacinas de que pode precisar?"
27 "... avaliar que exames de saúde precisa de fazer? (por ex. exame de mama, teste de açúcar no sangue, pressão sanguínea)"
28 "... avaliar se as informações sobre os riscos de saúde na mídia são de confiança? (por ex. TV, internet ou outros meios de comunicação)"
29 "... decidir se deve fazer a vacina contra a gripe?"
30 "... decidir como se pode proteger da doença com base nos conselhos da família e amigos?"
31 "... decidir como pode proteger-se da doença com base em informações oriundas dos meios de comunicação? (por ex. jornais, folhetos, internet ou outros meios de comunicação)"
32 "... encontrar informações sobre atividades saudáveis, tais como atividade física, alimentação saudável e nutrição?"
33 "... saber mais sobre as atividades que são boas para o seu bem-estar mental? (por ex. meditação, exercício, caminhadas, pilates, etc.)"
34 "... encontrar informações que indiquem como é que o seu bairro poderia ser mais amigo da saúde? (por ex. redução de ruído e poluição, criação de espaços verdes, de lazer)"
35 "... saber mais sobre as mudanças políticas que possam afetar a saúde? (por ex. legislação, programas de rastreio de saúde, novas mudanças de governo, de reestruturação de serviços de saúde, etc.)"
36 "... saber mais sobre os esforços para promover a sua saúde no trabalho?"
37 "... compreender conselhos sobre saúde que lhe chegam dos familiares ou amigos?"
38 "... compreender informação contida nas embalagens dos alimentos?"
26 "... avaliar quais são as vacinas de que pode precisar?"
27 "... avaliar que exames de saúde precisa de fazer? (por ex. exame de mama, teste de açúcar no sangue, pressão sanguínea)"
28 "... avaliar se as informações sobre os riscos de saúde na mídia são de confiança? (por ex. TV, internet ou outros meios de comunicação)"
29 "... decidir se deve fazer a vacina contra a gripe?"
30 "... decidir como se pode proteger da doença com base nos conselhos da família e amigos?"
31 "... decidir como pode proteger-se da doença com base em informações oriundas dos meios de comunicação? (por ex. jornais, folhetos, internet ou outros meios de comunicação)"

32 "... encontrar informações sobre atividades saudáveis, tais como atividade física, alimentação saudável e nutrição?"
33 "... saber mais sobre as atividades que são boas para o seu bem-estar mental? (por ex. meditação, exercício, caminhadas, pilates, etc.)"
34 "... encontrar informações que indiquem como é que o seu bairro poderia ser mais amigo da saúde? (por ex. redução de ruído e poluição, criação de espaços verdes, de lazer)"
35 "... saber mais sobre as mudanças políticas que possam afetar a saúde? (por ex. legislação, programas de rastreio de saúde, novas mudanças de governo, de reestruturação de serviços de saúde, etc.)"
36 "... saber mais sobre os esforços para promover a sua saúde no trabalho?"
37 "... compreender conselhos sobre saúde que lhe chegam dos familiares ou amigos?"
38 "... compreender informação contida nas embalagens dos alimentos?"
39 "... compreender a informação oriunda dos meios de comunicação sobre a forma de se tornar mais saudável?" (por ex. internet, jornais, revistas)"
40 "... compreender a informação que visa manter a mente saudável?"
41 "... avaliar como o local onde vive afeta a sua saúde e bem-estar?" (por ex. a sua comunidade, o seu bairro)
42 "... avaliar como as suas condições de habitação ajudam a permanecer saudável?"
43 "... avaliar que comportamento diário está relacionado com a sua saúde? (por ex. beber álcool, hábitos alimentares, exercício, etc.)"
44 "... tomar decisões para melhorar a sua saúde?"
45 "... ser sócio de um clube, praticar esportes ou aula de ginástica
46 "... influenciar as condições da sua vida que afetam a sua saúde e bem-estar? (por ex. ingestão de álcool, hábitos alimentares, exercício etc.)"
47"... tomar parte nas atividades que melhoram a saúde e o bem-estar na sua comunidade?"
39 "... compreender a informação oriunda dos meios de comunicação sobre a forma de se tornar mais saudável?" (por ex. internet, jornais, revistas)"
40 "... compreender a informação que visa manter a mente saudável?"
41 "... avaliar como o local onde vive afeta a sua saúde e bem-estar?" (por ex. a sua comunidade, o seu bairro)
42 "... avaliar como as suas condições de habitação ajudam a permanecer saudável?"
43 "... avaliar que comportamento diário está relacionado com a sua saúde? (por ex. beber álcool, hábitos alimentares, exercício, etc.)"
44 "... tomar decisões para melhorar a sua saúde?"
45 "... ser sócio de um clube, praticar esportes ou aula de ginástica
46 "... influenciar as condições da sua vida que afetam a sua saúde e bem-estar? (por ex. ingestão de álcool, hábitos alimentares, exercício etc.)"
47"... tomar parte nas atividades que melhoram a saúde e o bem-estar na sua comunidade?"

3) Demographic information

- Qual é o seu gênero *

1) Masculino
2) Feminino
3) Não quero responder

- Qual a sua data de nascimento? *

- Qual seu estado? *

1) Acre
2) Alagoas
3) Amapá
4) Amazonas

5) Bahia
6) Ceará
7) Distrito Federal
8) Espírito Santo
9) Goiás
10) Maranhão
11) Mato Grosso
12) Mato Grosso do Sul
13) Minas Gerais
14) Paraná
15) Paraíba
16) Pará
17) Pernambuco
18) Piauí
19) Rio Grande do Norte
20) Rio Grande do Sul
21) Rio de Janeiro
22) Rondônia
23) Roraima
24) Santa Catarina
25) Sergipe
26) São Paulo
27) Tocantins

10- Qual é o nível de educação mais elevado que concluiu com êxito (geralmente através da obtenção de um certificado ou diploma)? *

1) Nível 0 (pré-escolar/educação infantil/ sem finalização)
2) Nível 1 (Ensino fundamental I ou primeiro nível da educação básica)
3) Nível 2 (Educação fundamental II ou segunda etapa da educação básica)
4) Nível 3 (ensino médio ou ensino secundário)
5) Nível 4 (curso técnico ou tecnológico)
6) Nível 5 (ensino superior - graduação)
7) Nível 6 (pós-graduação lato sensu/especialização)
8) Nível 7 (pós-graduação stricto sensu - Mestrado, Doutorado, pósdoutorado e MBA)
9) não sei ler ou escrever
10) Não responde

Por favor, selecione agora o botão "enviar" ou "submit" para submeter o seu questionário. Muito obrigado pela sua participação!
