# Supplementary material for: Psychometric properties of the health literacy instrument in Brazil (HLS-EU-BR47)
Source: BMC Public Health. 2024 Jun 20;24:1655. doi: 10.1186/s12889-024-19108-2 (PMC11191178; doi:10.1186/s12889-024-19108-2)
Supplement: Supplementary file 3 — Supplementary Material 3 [file 12889_2024_19108_MOESM3_ESM.pdf]

APPENDIX A_English

1) Instrument introduction and informed consent

2) Health Literacy (HL) perception, measured with an instrument with 47 items described below (in English)

3) Demographic information

1) Instrument introduction and informed consent in English

Health literacy for all the voice of the community in the BiblioSUS network
The research aims to answer the question: How do people in the community express their information needs and the level of health literacy related to disease, quality of life and health promotion within the BiblioSUS Network?
This research constitutes a quali-quantitative study with the application of the online survey: Literacy for Health (HLS-EU-BR).

Thank you for your participation. We are available at: capagiic@ufrgs.br.

LEIA Research Health Literacy Network

* Indicates required question

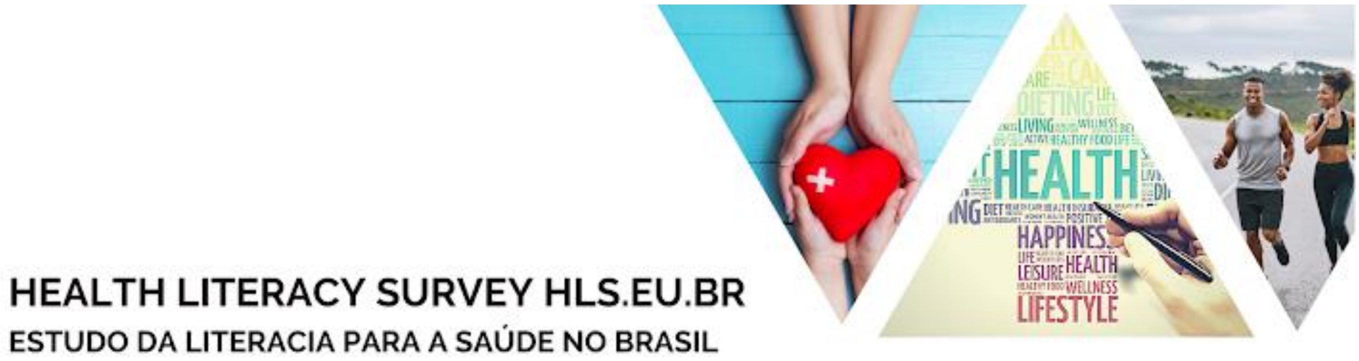

TERMS OF INFORMED CONSENT (TCLE) ONLINE SURVEY*
We invite you to voluntarily participate in the online survey of the Research Project "Health Literacy: the voice of the community in the BiblioSUS Network", carried out by the Interinstitutional Research Group LEIA: Reading, Information and Accessibility and by the Lusophone Health Literacy Network .Prof. Dr. Eliane Lourdes da Silva Moro is the researcher responsible, under the adjunct coordination of Prof. Dr. Lizandra Brasil Estabel and is registered within the CEP/UFRGS. The estimated time to answer all the questions is approximately 30 minutes. The study aims to answer the question: How do people in the community express their information needs and the level of health literacy related to disease, quality of life and health promotion within the BiblioSUS Network? The objectives are to identify the information needs and the level of health literacy related to disease, the quality of life and the health promotion of the community in accessing and using the BiblioSUS network and analyzing community needs in relation to Technologies of Information and Communication (ICT). The data collected by this online survey will be used for academic purposes, in partnership between the Federal University of Rio Grande do Sul (UFRGS) and the Ministry of

Health, preserving its confidentiality and guaranteeing the secrecy of the information collected. Thank you for the information provided. We are available for any clarification.

I agree to participate in the survey

I do not agree to participate in the survey

There are no right or wrong answers. The results obtained are confidential and used for purely statistical purposes. Please indicate the time when you will start filling out the survey:

2) **Health Literacy (HL) perception, measured with an instrument with 47 items described below (In English)**

(Health literacy dimension: diseases care)

1…find information about symptoms of illnesses that concern you?
2…find information on treatments of illnesses that concern you?
3…find out what to do in case of a medical emergency?
4…find out where to get professional help when you are ill? (e.g., from a doctor, pharmacist, psychologist)
5…understand what your doctor tells you?
6…understand the leaflets that come with your medicine?
7…understand what to do in a medical emergency?
8…understand your doctor’s or pharmacist’s instruction on how to take a prescribed medicine
9…judge how information from your doctor applies to you?
10…judge the advantages and disadvantages of different treatment options?
11…judge when you may need to get a second opinion from another doctor?
12…judge if the information about illness in the media is reliable? (e.g., TV, Internet or other media)
13…use information the doctor gives you to make decisions about your illness?
14…follow the instructions on medication?
15…call an ambulance in case of an emergency?
16…follow instructions from your doctor or pharmacist?

(Health literacy dimension: diseases prevention)

17…find information about how to manage unhealthy behaviour such as smoking, insufficient physical activity and drinking too much alcohol?
18…find information on how to manage mental health problems such as stress or depression?
19…find information about vaccinations and health screenings that you should have? (e.g., breast examination, blood sugar test, blood pressure)
20…find information on how to prevent or manage conditions such as being overweight, high blood pressure or high cholesterol?
21…understand health warnings about behaviour such as smoking, low physical activity and drinking too much?
22…understand why you need vaccinations?
23…understand why you need health screenings? breast exam, blood sugar test, blood pressure
24…judge how reliable health warnings are, such as smoking, low physical activity and drinking too much?
25…judge when you need to go to a doctor for a check-up?
26…judge which vaccinations you may need?

27…judge which health screenings you should have? (e.g., breast examination, blood sugar test, blood pressure)
28…judge if the information on health risks in the media is reliable? (e.g., TV, Internet or other media)
29…decide if you should have a flu vaccination?
30…decide how you can protect yourself from illness based on advice from family and friends?
31…decide how you can protect yourself from illness based on information in the media? (e.g., newspapers, leaflets, Internet or other media)

(Health literacy dimension: health promotion)

32…find information on healthy activities such as physical activity, healthy eating and nutrition?
33…find out about activities that are good for your mental well-being? (e.g., meditation, exercise, walking, pilates)
34…find information on how your neighbourhood could be more health-friendly? (e.g., reducing noise and pollution, creating green spaces, leisure facilities)
35…find out about policy changes that may affect health? (e.g., legislation, new health screening programmes, changing of government, restructuring of health services etc.)
36…find out about efforts to promote your health at work?
37…understand advice on health from family members or friends?
38 …understand information on food packaging?
39…understand information in the media on how to get healthier? (e.g., Internet, newspapers, magazines)
40…understand information on how to keep your mind healthy?
41…judge how where you live affects your health and well-being? (e.g., your community, your neighbourhood)
42…judge how your housing conditions help you to stay healthy?
43…judge which everyday behaviour is related to your health? (e.g., drinking and eating habits, exercise)
44…make decisions to improve your health?
45… join a sports club or exercise class if you want to?
46…influence your living conditions that affect your health and well being? (e.g., drinking and eating habits, exercise)
47…take part in activities that improve health and well-being in your community?

3) Demographic information

- What is your gender *

1) Male
2) Female
3) I don't want to answer

- What is your date of birth? *

- What is your state? *

1) Acre

2) Alagoas
3) Amapá
4) Amazonas
5) Bahia
6) Ceará
7) Distrito Federal
8) Espírito Santo
9) Goiás
10) Maranhão
11) Mato Grosso
12) Mato Grosso do Sul
13) Minas Gerais
14) Paraná
15) Paraíba
16) Pará
17) Pernambuco
18) Piauí
19) Rio Grande do Norte
20) Rio Grande do Sul
21) Rio de Janeiro
22) Rondônia
23) Roraima
24) Santa Catarina
25) Sergipe
26) São Paulo
27) Tocantins

10- What is the highest level of education you have successfully completed (usually by obtaining a certificate or diploma)? *

1) Level 0 (preschool/early childhood education/ without finalization)
2) Level 1 (Elementary School I or first level of basic education)
3) Level 2 (Elementary Education II or second stage of basic education)
4) Level 3 (high school or secondary school)
5) Level 4 (technical or technological course)
6) Level 5 (higher education - graduation)
7) Level 6 (lato sensu graduate/specialization)
8) Level 7 (stricto sensu postgraduate - Master's, Doctorate, Postdoctoral and MBA)
9) I can't read or write
10) Does not respond

Please now select the "send" or "submit" button to submit your questionnaire. Thank you for your participation!
