# Supplementary material for: Psychometric properties of the health literacy instrument in Brazil (HLS-EU-BR47)
Source: BMC Public Health. 2024 Jun 20;24:1655. doi: 10.1186/s12889-024-19108-2 (PMC11191178; doi:10.1186/s12889-024-19108-2)
Supplement: Supplementary file 4 — Supplementary Material 4 [file 12889_2024_19108_MOESM4_ESM.docx]

| **Items** | **Factor1** | **Communalities** |
| --- | --- | --- |
| HLHC01 | 0.59 | 0.35 |
| HLHC02 | 0.61 | 0.37 |
| HLHC03 | 0.67 | 0.45 |
| HLHC04 | 0.71 | 0.50 |
| HLHC05 | 0.80 | 0.65 |
| HLHC06 | 0.78 | 0.60 |
| HLHC07 | 0.68 | 0.46 |
| HLHC08 | 0.81 | 0.66 |
| HLHC09 | 0.79 | 0.63 |
| HLHC10 | 0.78 | 0.61 |
| HLHC11 | 0.74 | 0.55 |
| HLHC12 | 0.74 | 0.55 |
| HLHC13 | 0.72 | 0.51 |
| HLHC14 | 0.74 | 0.55 |
| HLHC15 | 0.66 | 0.43 |
| HLHC16 | 0.76 | 0.58 |
| HLDP17 | 0.71 | 0.51 |
| HLDP18 | 0.78 | 0.61 |
| HLDP19 | 0.80 | 0.63 |
| HLDP20 | 0.82 | 0.66 |
| HLDP21 | 0.80 | 0.64 |
| HLDP22 | 0.78 | 0.61 |
| HLDP23 | 0.81 | 0.66 |
| HLDP24 | 0.84 | 0.70 |
| HLDP25 | 0.81 | 0.65 |
| HLDP26 | 0.74 | 0.54 |
| HLDP27 | 0.81 | 0.65 |
| HLDP28 | 0.72 | 0.52 |
| HLDP29 | 0.76 | 0.57 |
| HLDP30 | 0.69 | 0.48 |
| HLDP31 | 0.74 | 0.55 |
| HLHP32 | 0.80 | 0.64 |
| HLHP33 | 0.79 | 0.62 |
| HLHP34 | 0.53 | 0.28 |
| HLHP35 | 0.64 | 0.41 |
| HLHP36 | 0.60 | 0.36 |
| HLHP37 | 0.72 | 0.52 |
| HLHP38 | 0.74 | 0.55 |
| HLHP39 | 0.77 | 0.60 |
| HLHP40 | 0.80 | 0.64 |
| HLHP41 | 0.69 | 0.48 |
| HLHP42 | 0.75 | 0.56 |
| HLHP43 | 0.70 | 0.49 |
| HLHP44 | 0.62 | 0.38 |
| **HLHP45** | **0.53** | **0.29** |
| HLHP46 | 0.60 | 0.36 |
| HLHP47 | 0.54 | 0.29 |
